# Multi-layer gut microbiome variation in type 2 diabetes despite preserved higher-order community structure

**DOI:** 10.3389/fmicb.2026.1902019

**Published:** 2026-08-14

**Authors:** Junwei Zhou, Peijie Du, Limin Liu, Li Wang, Suping Liu, Guizhi Yan

**Affiliations:** 1 Department of Biochemistry and Molecular Biology, Shangqiu Medical College, Shangqiu, China; 2 Department of Endocrinology and Metabolic Diseases, The First Affiliated Hospital of Zhengzhou University, Zhengzhou, China; 3 The Second Affiliated Hospital of Shangqiu Medical College, Shangqiu, China

**Keywords:** gut microbiome, microbial architecture, microbial co-occurrence, systems microbiology, type 2 diabetes

## Abstract

**Background:**

Type 2 diabetes (T2D) has been consistently associated with alterations in the gut microbiome, although disease, treatment, diet, and other host factors may contribute to the observed patterns. How these associations are organized across different biological levels of the microbial ecosystem remains incompletely understood.

**Methods:**

We performed shotgun metagenomic sequencing of fecal samples from 82 individuals, including 41 patients with T2D and 41 age-, sex-, and body mass index-matched healthy controls. Taxonomic profiling, functional pathway analysis, enterotype characterization, ecological network inference, and interpretable machine-learning approaches were integrated to characterize microbiome variation across multiple organizational levels.

**Results:**

Despite clear clinical differences between groups, particularly fasting blood glucose, the overall ecological architecture of the gut microbiome remained broadly preserved. Dominant phylum-level composition and enterotype structure were maintained, whereas variation became apparent at finer biological scales. Species-level analyses identified 42 differentially abundant taxa. Community diversity analysis showed reduced Chao1 richness (*P* = 0.024), increased Simpson diversity (*P* = 0.024), unchanged Shannon diversity (*P* = 0.126), and a modest shift in community composition (PERMANOVA, *R*^2^ = 0.040, *P* = 0.006). Functional profiling showed no pathway-level significance after multiple-testing correction but directional trends across several metabolic modules. Exploratory Spearman-based networks differed in topology between groups; because relative-abundance data are compositional, these differences cannot be interpreted as direct ecological interactions or definitive network rewiring. Machine-learning models achieved a within-cohort cross-validated AUC of up to 0.91, but lacked independent external validation.

**Conclusions:**

These findings provide a multi-layer description of T2D-associated gut microbiome variation within this cohort. The data are consistent with preserved higher-order community organization accompanied by finer-scale differences in species composition, functional potential, community-state occupancy, statistical co-occurrence, and within-cohort discriminative features. Medication confounding, compositional effects, technical artifacts, and the absence of external validation limit causal, ecological, and diagnostic interpretation. Larger longitudinal, multi-site, medication-resolved, and independently validated studies are required.

## Graphical Abstract

**Figure:**
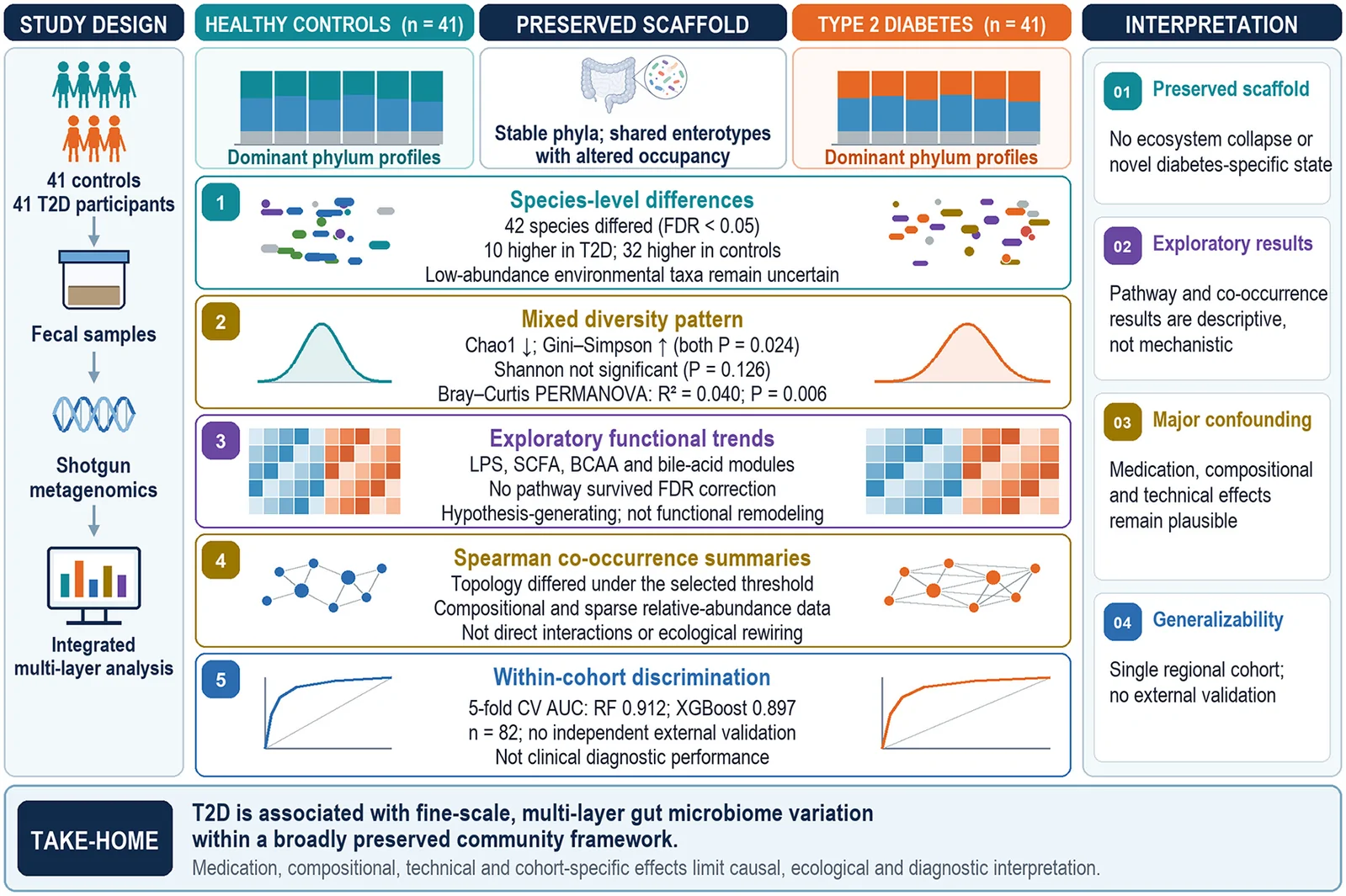

## 1 Introduction

Type 2 diabetes (T2D) is a complex metabolic disorder characterized by chronic hyperglycemia resulting from insulin resistance and progressive pancreatic β-cell dysfunction (Wang et al., 2020; Magliano and Boyko, 2025). As one of the fastest-growing non-communicable diseases worldwide, T2D imposes a substantial burden on public health systems and is associated with increased risks of cardiovascular disease, kidney dysfunction, neuropathy, and premature mortality (Wang et al., 2020). Although host genetics, dietary habits, lifestyle factors, and environmental exposures contribute to disease susceptibility, these factors alone do not fully explain the marked heterogeneity observed in T2D onset, progression, and metabolic phenotypes (Ghatan et al., 2024; Suzuki et al., 2024).

Over the past decade, the gut microbiome has emerged as a potential contributor to metabolic homeostasis and T2D pathophysiology (Cani et al., 2007; Qin et al., 2012; Karlsson et al., 2013; Song et al., 2025; Wu et al., 2025; Gurung et al., 2020). Microbial metabolites provide several plausible host-facing routes: short-chain fatty acids can influence epithelial integrity, enteroendocrine signaling, and peripheral insulin sensitivity; microbially modified bile acids signal through FXR and TGR5; and branched-chain amino acids and other microbial products may track or modulate metabolic dysfunction (Song et al., 2025; Gurung et al., 2020; Canfora et al., 2019; Morrison and Preston, 2016; Wahlstrom et al., 2016). In parallel, impaired barrier integrity may increase exposure to lipopolysaccharide and other microbial products, promoting innate immune activation, low-grade inflammation, and insulin resistance (Cani et al., 2007; Gurung et al., 2020; Cani and Jordan, 2018). These metabolic, immune, and barrier pathways are interconnected and bidirectional, because hyperglycemia, diet, adiposity, and treatment can also reshape the intestinal habitat. Accordingly, reported differences in diversity, taxonomic composition, and functional potential in T2D should be interpreted as components of a host–microbiome system rather than isolated microbial causes (Qin et al., 2012; Karlsson et al., 2013; Wu et al., 2025; Gurung et al., 2020).

Although the present study focuses on fecal communities, a systemic microecological perspective also requires consideration of spatially connected body sites. T2D-associated dysbiosis has been reported concurrently in oral and intestinal communities, with shared taxa, correlations with inflammatory indices, and a measurable but limited contribution of oral sources to fecal communities (Guo et al., 2023). The oral–gut axis could influence metabolic inflammation through periodontal barrier disruption, swallowed microbial products, and context-dependent ectopic colonization. Conversely, hyperglycemia, medication, diet, and immune dysfunction can affect both niches. Multi-site longitudinal sampling is therefore needed to determine whether oral and gut changes are coordinated components of diabetes-associated ecology rather than parallel consequences of the same host exposures.

Despite these advances, the biological interpretation of T2D-associated microbiome alterations remains incomplete. Most previous studies have focused primarily on differential abundance analyses of individual taxa or pathways, aiming to identify specific microorganisms associated with disease status (Qin et al., 2012; Karlsson et al., 2013). While such approaches have generated valuable insights, they have also produced considerable inconsistency across cohorts and populations, with relatively limited overlap among reported microbial biomarkers (Qin et al., 2012; Lloyd-Price et al., 2017). This inconsistency suggests that disease-associated microbiome alterations may not be fully explained by changes in individual microorganisms alone.

An emerging perspective in microbial ecology is that the gut microbiome functions as a complex adaptive ecosystem rather than a simple collection of independent taxa (Lloyd-Price et al., 2017; Faust and Raes, 2012; Faust et al., 2012; Prosser et al., 2007). Within such ecosystems, microbial functions arise from interactions among community members, including competition, cooperation, metabolic cross-feeding, and ecological niche partitioning. Consequently, disease-associated microbial alterations may reflect coordinated changes in ecosystem organization, functional capacity, and interaction architecture rather than isolated compositional abnormalities (Faust and Raes, 2012; Faust et al., 2012; Prosser et al., 2007; Berry and Widder, 2014).

Ecological theory predicts that environmental perturbations can induce ecosystem reconfiguration across multiple organizational layers, including species composition, metabolic functions, interaction networks, and community-state occupancy, without necessarily disrupting higher-order ecosystem structure (Faust and Raes, 2012; Faust et al., 2012; Prosser et al., 2007). Under this framework, disease-associated dysbiosis may emerge as a systems-level property characterized by progressive ecological reorganization rather than large-scale ecosystem collapse. Such a perspective has important implications for understanding host–microbiome interactions because microbial community behavior may be governed by collective ecosystem properties that are not readily apparent from taxonomic composition alone (Berry and Widder, 2014; Banerjee et al., 2018; Louca et al., 2018).

Recent advances in shotgun metagenomic sequencing provide an opportunity to investigate these questions at unprecedented resolution (Abubucker et al., 2012; Segata et al., 2012). Compared with 16S rRNA gene sequencing, shotgun metagenomics enables simultaneous characterization of microbial composition and functional metabolic potential, allowing ecosystem organization to be examined across multiple biological scales (Quince et al., 2017). However, the interpretation of high-dimensional metagenomic data remains challenging, particularly when attempting to integrate taxonomic variation, functional remodeling, ecological interactions, and disease-associated phenotypes into a coherent biological framework.

To address these challenges, systems-level analytical approaches have increasingly been incorporated into microbiome research. Ecological network analysis enables investigation of microbial interaction architecture and community organization beyond simple abundance profiles (Faust and Raes, 2012; Faust et al., 2012). Alterations in network topology, modular structure, and connectivity may reveal changes in ecosystem resilience and ecological integration associated with disease states (Berry and Widder, 2014; Coyte et al., 2015). In parallel, machine-learning approaches have emerged as powerful tools for identifying complex microbial signatures and improving disease classification (Acharjee, 2023; Novielli et al., 2024). Nevertheless, many predictive models remain difficult to interpret biologically because they rely on black-box computational frameworks. Interpretable machine-learning methods, such as SHapley Additive exPlanations (SHAP), provide an opportunity to identify biologically meaningful contributors to predictive performance while preserving analytical transparency (Lundberg and Lee, 2017; Dai et al., 2026).

In the present study, we investigated gut microbiome alterations associated with T2D using an integrated analytical framework that combined shotgun metagenomic sequencing, functional pathway profiling, enterotype characterization, ecological network analysis, and interpretable machine learning in a Chinese Han cohort. Rather than focusing solely on differential microbial abundance, we sought to examine whether taxonomic composition, functional potential, community-state organization, microbial interaction architecture, and discriminative microbial signatures exhibit coordinated patterns across multiple organizational levels. By integrating these complementary analyses within a single cohort, we aimed to provide a systems-level characterization of gut microbiome organization in T2D while placing our findings in the context of previous microbiome studies.

Our analyses evaluate multiple analytical layers of microbiome variation associated with T2D, including species-level composition, functional trends, enterotype occupancy, statistical co-occurrence structure, and within-cohort discriminative signatures. Collectively, these observations provide a systems-level descriptive framework. They do not establish causal ecological remodeling, direct microbial interactions, or a clinically generalizable diagnostic signature.

## 2 Materials and methods

### 2.1 Study cohort and study design

A total of 82 participants were enrolled in this case–control study, including 41 patients with T2D and 41 healthy controls with broadly comparable age, sex, and BMI distributions. The two groups were comparable in demographic characteristics, whereas fasting blood glucose levels were significantly higher in the T2D group (*P* < 0.01), confirming group stratification.

T2D diagnosis was established according to internationally recognized clinical criteria (American Diabetes Association Professional Practice Committee, 2024). Healthy controls had no history of diabetes or other major metabolic disorders. Participants were excluded if they had used antibiotics, probiotics, or prebiotics within 3 months prior to sample collection, had undergone gastrointestinal surgery, or had a history of chronic gastrointestinal disease, acute infection, malignancy, or severe hepatic, renal, or systemic disorders.

All participants provided written informed consent. The study protocol was approved by the ethics committee of Shangqiu Medical College and conducted in accordance with the Declaration of Helsinki. All samples were processed using standardized protocols for DNA extraction, library preparation, and sequencing to minimize technical variation.

### 2.2 Fecal sample collection and microbial DNA extraction

Fresh fecal samples were collected using sterile tubes containing DNA stabilization buffer (0.5 M EDTA, 0.5 M Tris-HCl, and 2 M guanidine thiocyanate). Samples were transported on ice, aliquoted immediately upon receipt, and stored at −25 °C until DNA extraction. Microbial genomic DNA was extracted using the Tiangen Stool DNA Kit (DP328, China) according to the manufacturer's instructions, combining mechanical disruption and chemical lysis followed by silica membrane purification. DNA concentration and integrity were assessed using NanoDrop spectrophotometry, Qubit fluorometry, and agarose gel electrophoresis. Only samples passing quality control were included in downstream analyses.

### 2.3 Shotgun metagenomic sequencing

Metagenomic libraries were prepared from approximately 0.2 μg of genomic DNA per sample. DNA was fragmented to an average insert size of ~350 bp, followed by end repair, A-tailing, adapter ligation, and PCR amplification. Library quality was assessed using an Agilent 5400 Bioanalyzer and quantitative PCR. Paired-end sequencing (150 bp) was performed on the DNBSEQ-T7 platform (MGI, China), generating high-depth metagenomic datasets.

### 2.4 Sequence quality control and host read removal

Raw sequencing reads were processed using fastp (v0.23.1) for adapter trimming and quality filtering. Reads containing >10% ambiguous bases or >50% low-quality bases (Phred score < 5) were removed. Host-derived reads were removed by aligning reads to the human reference genome (GRCh38) using Bowtie2 (v2.4.4), and aligned reads were discarded.

### 2.5 Taxonomic and functional profiling of the gut microbiome

#### 2.5.1 Taxonomic profiling

Taxonomic profiling was performed using Kraken2 (v2.1.3) with the Genome Taxonomy Database (GTDB, release R207) as reference (Parks et al., 2020; Lu et al., 2017). Abundance refinement was performed using Bracken (v2.6.2). Taxonomic profiles were generated at phylum, genus, and species levels.

#### 2.5.2 Functional profiling

Functional annotation was performed using HUMAnN3 (v3.6) (Beghini et al., 2021). Gene families were first mapped to UniRef90, and pathway reconstruction was conducted using the MetaCyc database (v25) (Caspi et al., 2020). Pathway abundance outputs were normalized within HUMAnN3 to account for gene length and sequencing depth, and further expressed as either relative abundance or copies-per-million (CPM)-scaled values depending on downstream analysis requirements. For consistency in comparative analyses, CPM-normalized pathway abundance matrices were used for feature filtering and visualization.

### 2.6 Feature preprocessing and ecological filtering

To reduce sparsity and improve robustness of downstream analyses, taxonomic, and functional profiles were subjected to a unified ecological filtering strategy. Features annotated as “UNMAPPED” or “UNINTEGRATED,” as well as viral, phage-associated, and low-confidence artifacts, were excluded. In addition, features detected in fewer than 5% of samples were removed. Low-abundance features were filtered using scale-specific thresholds. For taxonomic features, taxa with mean relative abundance < 1 × 10^−6^ were excluded. For functional pathways, features with mean abundance < 0.5 CPM were removed. These thresholds were applied according to the native abundance scale of each data type and were not directly comparable across feature classes.

All downstream statistical analyses were conducted on filtered abundance matrices, followed by appropriate transformation (e.g., log transformation or Z-score normalization) depending on the specific analysis. Following filtering, the final dataset comprised 8 phyla, 959 genera, 1,640 species, and 486 metabolic pathways.

### 2.7 Diversity and ecological structure analysis

#### 2.7.1 Alpha diversity

Alpha diversity was assessed from species-level abundance profiles using the Shannon index, Chao1 richness, and the Gini–Simpson index (1 – D). Rarefaction analysis was performed prior to diversity estimation to evaluate sequencing-depth sufficiency and sampling completeness across samples. Between-group differences in alpha-diversity metrics were assessed using two-sided Wilcoxon rank-sum tests.

#### 2.7.2 Beta diversity

Beta diversity was evaluated using Bray–Curtis dissimilarity. Principal coordinates analysis (PCoA) was used for ordination and visualization of between-sample compositional variation, and group differences were tested using permutational multivariate analysis of variance (PERMANOVA) with 999 permutations.

### 2.8 Differential abundance and functional analysis

#### 2.8.1 Taxonomic differential analysis

Differential abundance analysis at taxonomic levels was performed using a combination of log_2_ fold-change estimation and the non-parametric Wilcoxon rank-sum test. *P*-values were adjusted for multiple comparisons using the Benjamini–Hochberg false discovery rate (FDR) correction, with features considered significant at FDR < 0.05 and |log_2_ fold-change| ≥ 1.0.

#### 2.8.2 Functional differential analysis

Functional pathway differences were assessed using the same statistical framework. Although no pathways remained significant after FDR correction, consistent directional trends were evaluated across metabolic modules to support systems-level interpretation.

### 2.9 Microbiome–host association analysis

#### 2.9.1 Correlation analysis

Associations between microbial features and host clinical variables (age, BMI, fasting blood glucose, lipid profile, liver and renal function markers, and T2D status) were evaluated using Spearman correlation with FDR correction. These analyses used filtered relative-abundance profiles and were exploratory. Because such profiles are compositional, sparse, and zero-inflated, conventional rank correlations can contain induced or spurious associations and do not estimate independent taxon–taxon or taxon–host effects (Gloor et al., 2017; Kurtz et al., 2015).

#### 2.9.2 Multivariable regression analysis

Associations were further evaluated using multivariable linear regression models including age, BMI, fasting blood glucose, and T2D status as covariates. Microbial abundance data were log-transformed prior to model fitting.

### 2.10 Machine learning framework for disease classification

#### 2.10.1 Feature selection and leakage prevention

To prevent information leakage, all feature selection procedures were performed independently within each training fold of the cross-validation framework. Features were initially ranked using univariate ROC-AUC scores computed on training data only. All feature scaling and normalization steps were also restricted to training folds, and identical transformations were applied to validation sets.

#### 2.10.2 Predictive modeling

Two supervised learning models were implemented: Random Forest classifiers (100 trees, maximum depth = 4) and XGBoost classifiers (100 estimators, learning rate = 0.1, maximum depth = 4, subsampling ratio = 0.8, column sampling ratio = 0.8). These hyperparameters were selected based on prior microbiome classification studies and preliminary tuning.

#### 2.10.3 Model evaluation

Model performance was assessed using stratified five-fold cross-validation. The primary evaluation metric was ROC-AUC, with accuracy, precision, recall, and F1-score reported as secondary metrics. Mean performance and 95% confidence intervals were calculated across folds.

### 2.11 Interpretable machine learning

SHapley Additive exPlanations (SHAP) were used to interpret model outputs. TreeExplainer was applied to both Random Forest and XGBoost models. Global feature importance was quantified using mean absolute SHAP values, and local explanations were used to identify sample-specific microbial signatures associated with T2D.

### 2.12 Enterotype analysis

Enterotype classification was performed using genus-level profiles (Arumugam et al., 2011). Jensen–Shannon divergence (JSD) was used to compute pairwise distances, and Partitioning Around Medoids (PAM) clustering was applied. The optimal number of clusters was determined using silhouette and Calinski–Harabasz indices. Differences in enterotype distribution were assessed using Fisher's exact test.

### 2.13 Ecological network analysis

Species-level statistical co-occurrence networks were constructed separately for control and T2D samples using Spearman correlation analysis. Prior to network inference, low-prevalence species were excluded to reduce sparsity and unstable correlations. Pairwise associations were retained as edges when the absolute correlation coefficient was ≥0.6 and the false discovery rate (FDR)-adjusted *P*-value was < 0.05. No compositionality-aware network method was applied; consequently, closure, sparsity, and group-specific abundance distributions may generate or remove edges and may influence density, degree, and modularity (Gloor et al., 2017; Kurtz et al., 2015). The networks were therefore treated as exploratory summaries of covariance under this pipeline, not as estimates of ecological interaction networks.

Network topology was summarized using node number, edge number, network density, average degree, average clustering coefficient, and average shortest path length. Louvain community detection was performed on each group-specific absolute edge-weight graph, and degree and betweenness centrality were calculated. Because topology depends on the correlation estimator and threshold, between-group differences in these metrics were interpreted descriptively. Highly connected nodes are referred to as statistical hubs only and not as ecological keystone taxa.

### 2.14 Statistical analysis and reproducibility

All statistical analyses were performed using Python 3.7 with standard scientific computing libraries. Statistical significance was defined as FDR < 0.05 unless otherwise specified. To ensure reproducibility, a fixed random seed (42) was applied across all stochastic procedures, including machine learning model training and cross-validation splits. All scripts and analytical workflows were executed in a controlled computational environment to ensure consistency of results.

### 2.15 Integrated systems-level analysis

To characterize gut microbiome variation associated with T2D, analyses were conducted across multiple layers, including taxonomic composition, diversity, functional pathways, Spearman-based co-occurrence summaries, and within-cohort machine-learning signatures. This framework integrates complementary descriptive analyses but does not by itself establish coordinated ecological change or causal relationships.

## 3 Results

### 3.1 Cohort characteristics and sequencing overview

A total of 82 individuals were included in this study, comprising 41 patients with type 2 diabetes (T2D) and 41 age-, sex-, and body mass index (BMI)-matched healthy controls. The two groups were comparable in demographic characteristics, whereas fasting blood glucose levels were significantly elevated in the T2D cohort, confirming clear clinical separation between groups (Table 1; Supplementary Table 1).

**Table 1:** Baseline demographic and clinical characteristics of study participants.

Variable	Control (*n* = 41)	T2D (*n* = 41)	*P*-value
Age (years)	53.10 ± 5.88	54.24 ± 4.83	0.34
Male (*n*, %)	25 (61%)	22 (54%)	0.53
BMI (kg/m^2^)	24.66 ± 2.91	25.21 ± 3.48	0.45
Fasting glucose (mmol/L)	6.31 ± 2.86	8.54 ± 4.34	< 0.01

Shotgun metagenomic sequencing generated a high-depth dataset with a mean of 55.56 million reads per sample (Table 2). Following quality control and host-read removal, more than 99% of reads were retained across samples (Supplementary Figure 1), indicating high technical read quality and sufficient depth for downstream taxonomic and functional analyses. These read-level metrics do not, however, exclude reagent contamination or validate individual low-abundance taxonomic assignments.

**Table 2:** Summary of sequencing data.

Metric	Control (*n* = 41)	T2D (*n* = 41)
Raw reads (million)	53.29 ± 21.02	57.83 ± 12.34
Clean reads (million)	52.91 ± 20.21	57.54 ± 12.30
Q30 (%)	98.72 ± 0.001	97.76 ± 0.01
Host reads (%)	0.49 ± 1.26	0.48 ± 1.08

### 3.2 Global ecological architecture remains preserved in T2D

**Figure 1:**
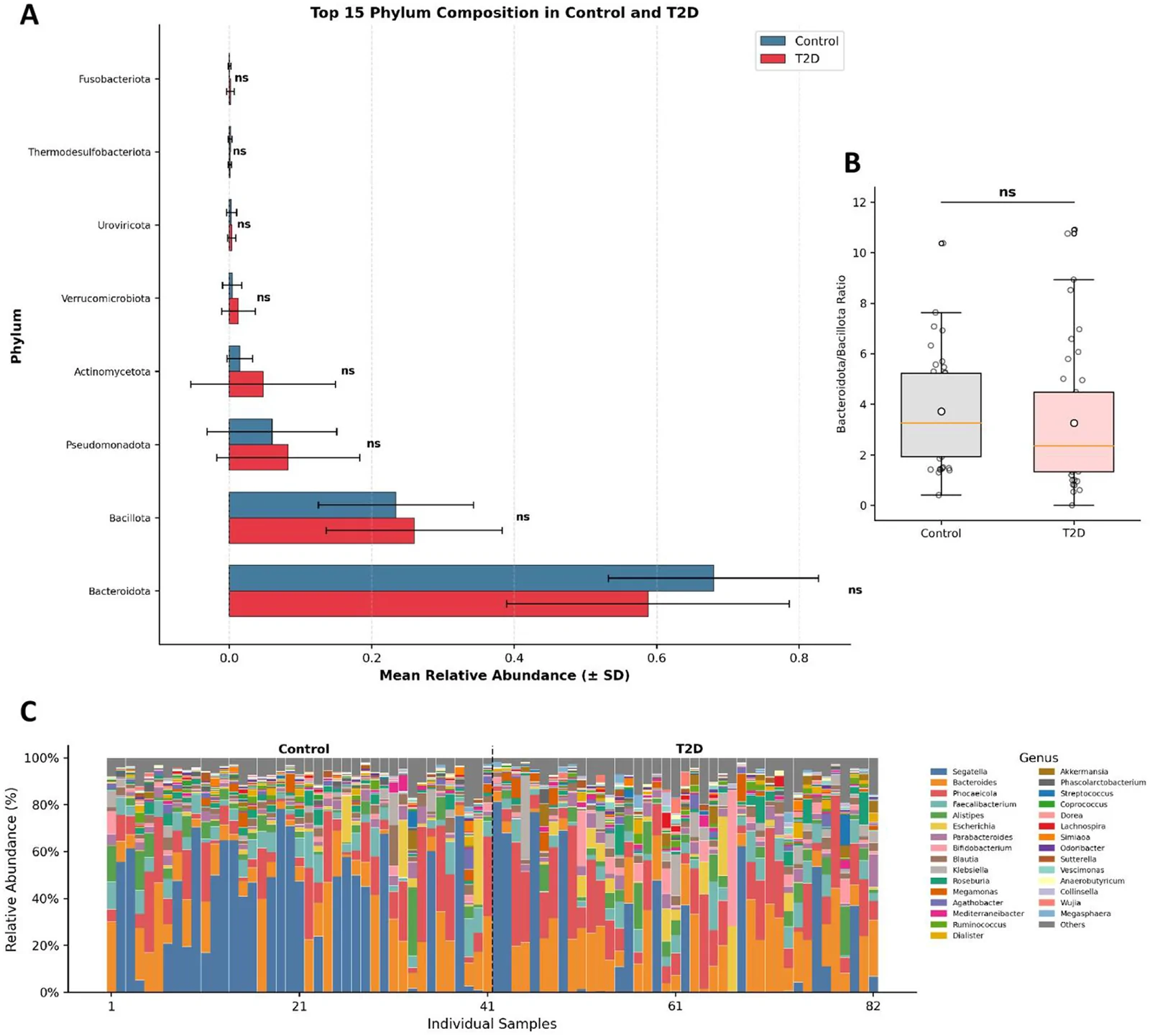
Overall taxonomic architecture remains largely stable in T2D despite subtle compositional shifts. **(A)** Relative abundance of dominant bacterial phyla in T2D and healthy control groups. **(B)** Comparison of the Bacteroidota-to-Bacillota ratio between groups. **(C)** Genus-level taxonomic composition across all study participants. Taxonomic profiles were generated from shotgun metagenomic sequencing data following abundance filtering. All analyses were based on total-sum scaled relative abundance profiles to ensure comparability across samples. Statistical significance was assessed using two-sided Wilcoxon rank-sum tests with Benjamini–Hochberg false discovery rate (FDR) correction. ns, not statistically significant.

Metagenomic profiling identified 128 bacterial phyla, 3,199 genera, and 14,068 species before abundance filtering. Following quality control, gut microbial communities in both groups were dominated by members of the phyla Bacteroidota and Bacillota (Figure 1A). Despite clear clinical differences between groups, phylum-level community composition remained remarkably stable. No significant differences were observed in the relative abundance of dominant phyla or in the Bacteroidota/Bacillota ratio (Figure 1B). Similarly, genus-level microbial profiles displayed substantial inter-individual variability but lacked obvious disease-specific clustering patterns (Figure 1C, Supplementary Figure 2).

Among 959 filtered genera, only two taxa, Aeromicrobium and Terribium, remained significantly different after multiple-testing correction (FDR < 0.05, |log_2_FC| ≥ 1) (Supplementary Figure 3; Supplementary Table 2). Nevertheless, effect-size distributions revealed a pronounced directional asymmetry, with 697 genera showing positive fold changes in T2D compared with 262 genera showing negative fold changes (Supplementary Table 2).

These observations indicate that T2D does not induce large-scale disruption of gut microbial community structure. Instead, higher-order ecological architecture remains largely preserved despite subtle directional compositional shifts.

### 3.3 Species-level differences include technically uncertain low-abundance taxa

**Figure 2:**
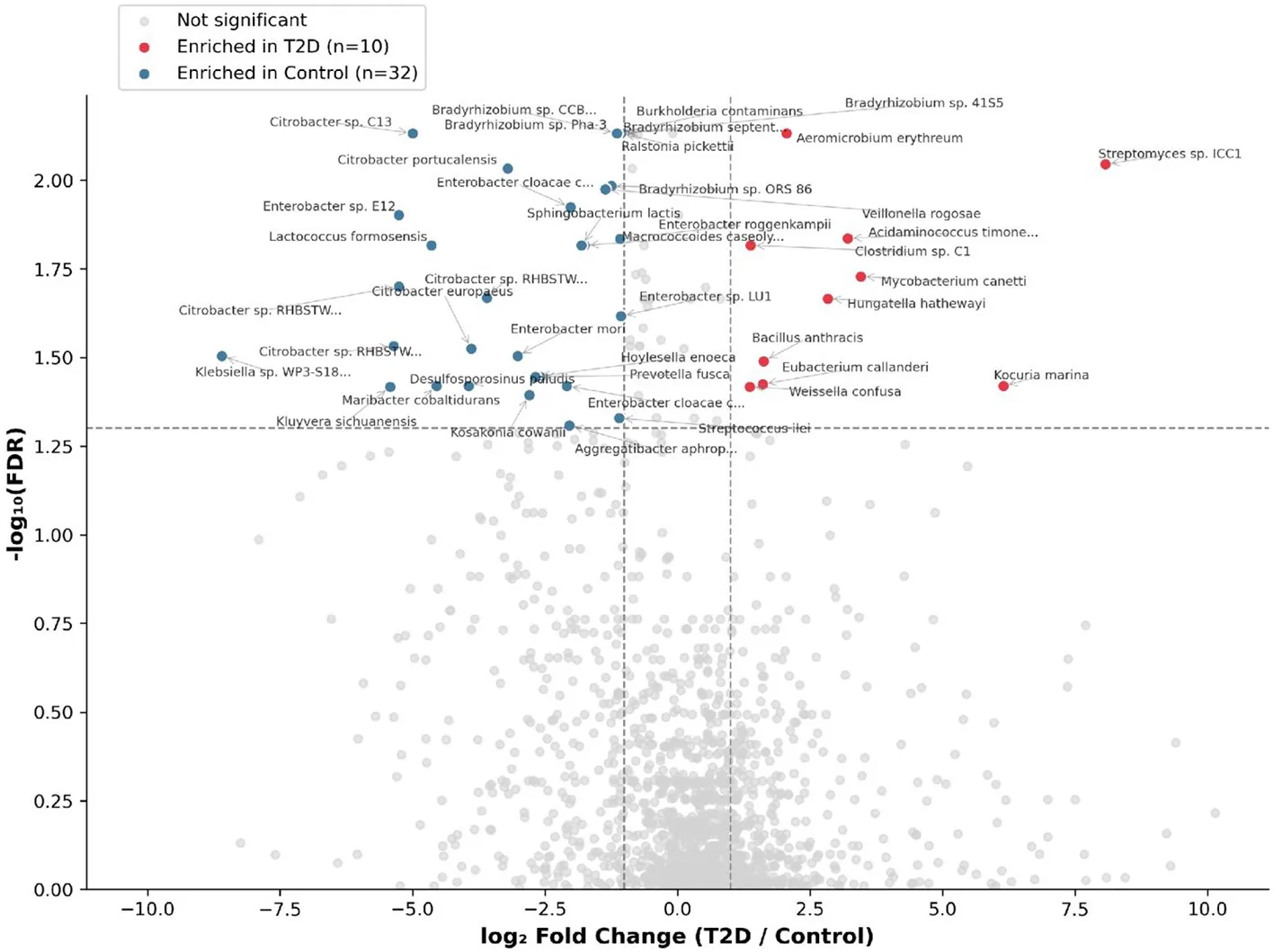
Volcano plot of microbial species associated with T2D. Volcano plot showing differential abundance analysis of 1,640 filtered microbial species between T2D and control groups.

Although phylum- and genus-level community structures were largely conserved, ecological divergence became increasingly apparent at species-level resolution. After abundance filtering, 1,640 species were retained for analysis. Differential abundance testing identified 42 significantly altered species (FDR < 0.05, |log_2_FC| ≥ 1), including 10 enriched in T2D and 32 enriched in healthy controls (Figure 2; Supplementary Table 3). The distribution of effect sizes among differentially abundant microbial species is shown in Supplementary Figure 4.

**Figure 3:**
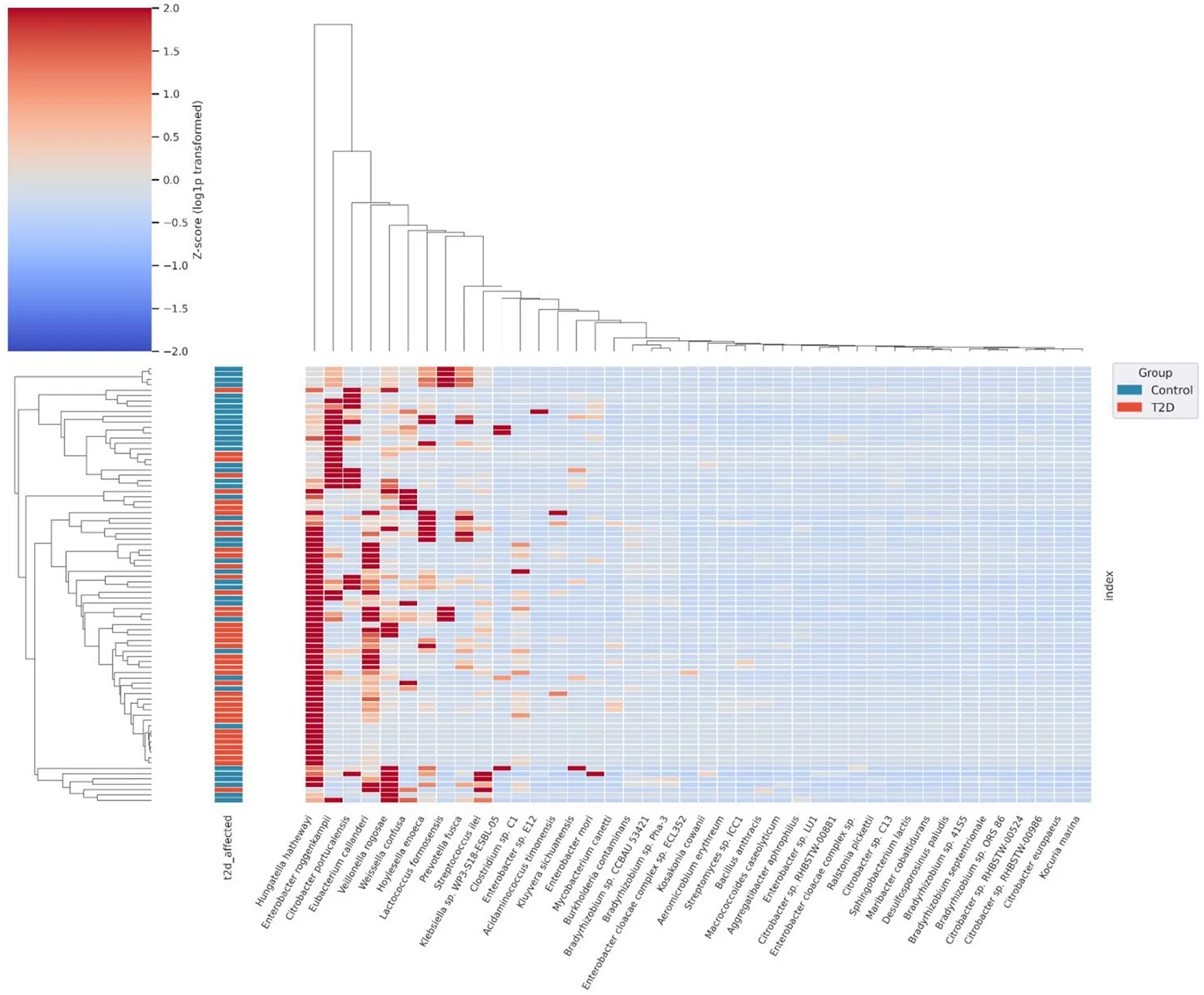
Heatmap of significantly altered microbial species. Heatmap showing the relative abundance patterns of 42 significantly altered microbial species (FDR < 0.05 and |log_2_FC| ≥ 1).

**Figure 4:**
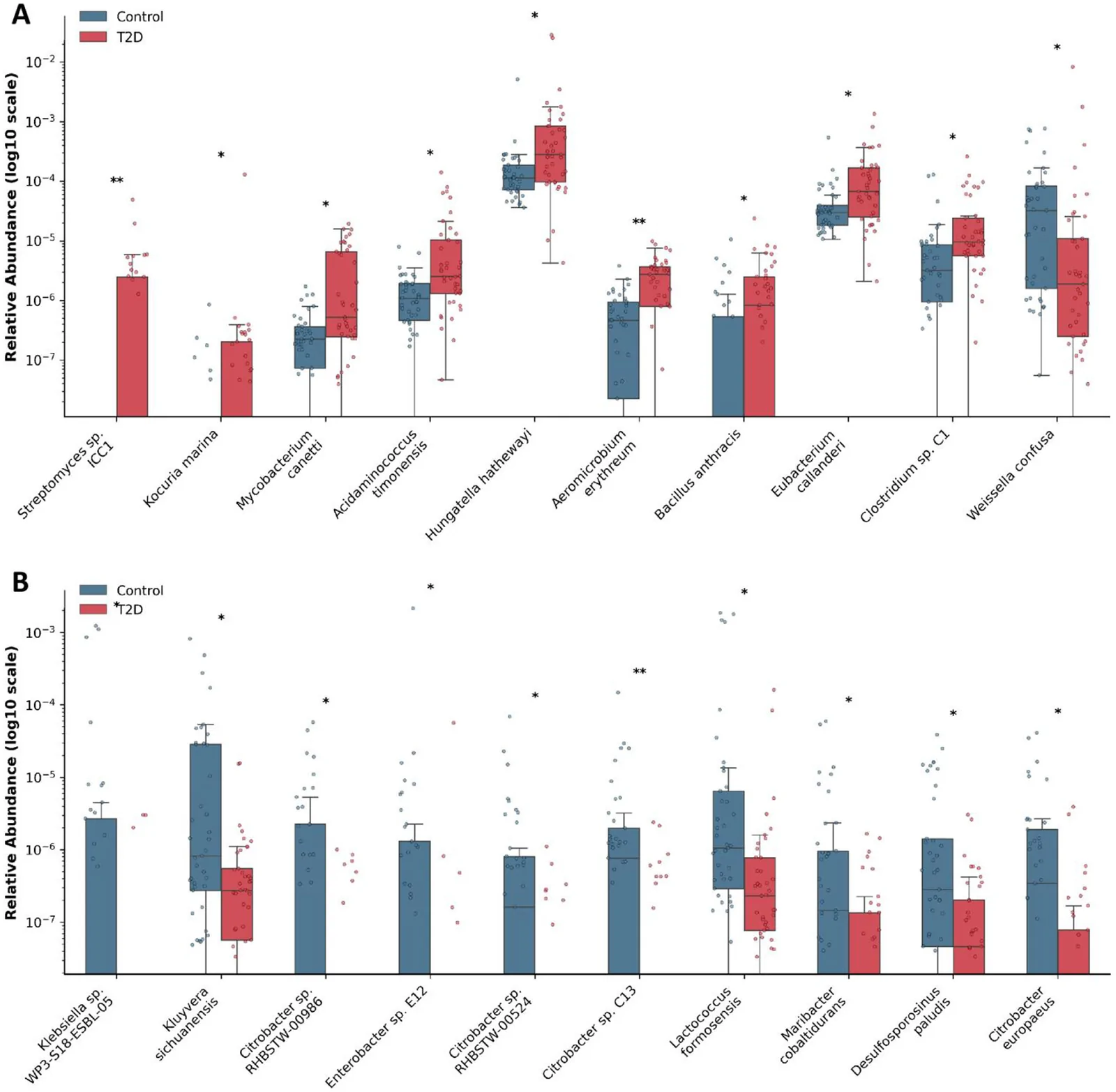
Differentially enriched microbial species in T2D and healthy controls. **(A)** Top 10 species enriched in T2D (log_2_FC ≥ 1, FDR < 0.05). **(B)** Top 10 species enriched in healthy controls (log_2_FC ≤ −1, FDR < 0.05). ^*^Indicates FDR-adjusted *P* < 0.05; ^**^indicates FDR-adjusted *P* < 0.01.

Volcano plot analysis demonstrated clear separation between discriminatory species and the background distribution (Figure 2). Heatmap visualization further revealed consistent abundance shifts across individuals (Figure 3). Aeromicrobium erythreum was enriched in T2D samples, whereas several *Bradyrhizobium-* and Citrobacter-related assignments were depleted (Figure 4). These statistical differences were concentrated within a relatively small subset of species-level features. However, several leading signals belonged to low-abundance, environmentally associated genera, including *Bradyrhizobium* and *Aeromicrobium*. Without extraction blanks, negative controls, or orthogonal taxonomic confirmation, background environmental DNA, reagent contamination, low-abundance classification uncertainty, reference-database misassignment, or a systematic batch artifact cannot be excluded (Salter et al., 2014). We therefore regard these features as technically uncertain signals rather than evidence of gut colonization or disease-relevant organisms.

Collectively, these findings suggest that T2D-associated microbial signatures arise from localized species-level reorganization embedded within an otherwise stable community framework.

### 3.4 Alpha-diversity metrics show mixed patterns and beta diversity differs modestly

We next assessed whether T2D was associated with broad disruptions in gut microbial diversity. Alpha-diversity analysis revealed selective rather than global shifts in within-sample diversity. Compared with controls, T2D subjects exhibited significantly lower Chao1 richness (*P* = 0.024), indicating reduced taxonomic richness, but significantly higher Simpson diversity (*P* = 0.024), consistent with a more even within-community abundance distribution. Shannon diversity remained comparable between groups (*P* = 0.126). Together, these findings suggest that T2D was associated with a redistribution of alpha-diversity structure across richness and evenness dimensions, rather than a uniform depletion of microbial diversity.

**Figure 5:**
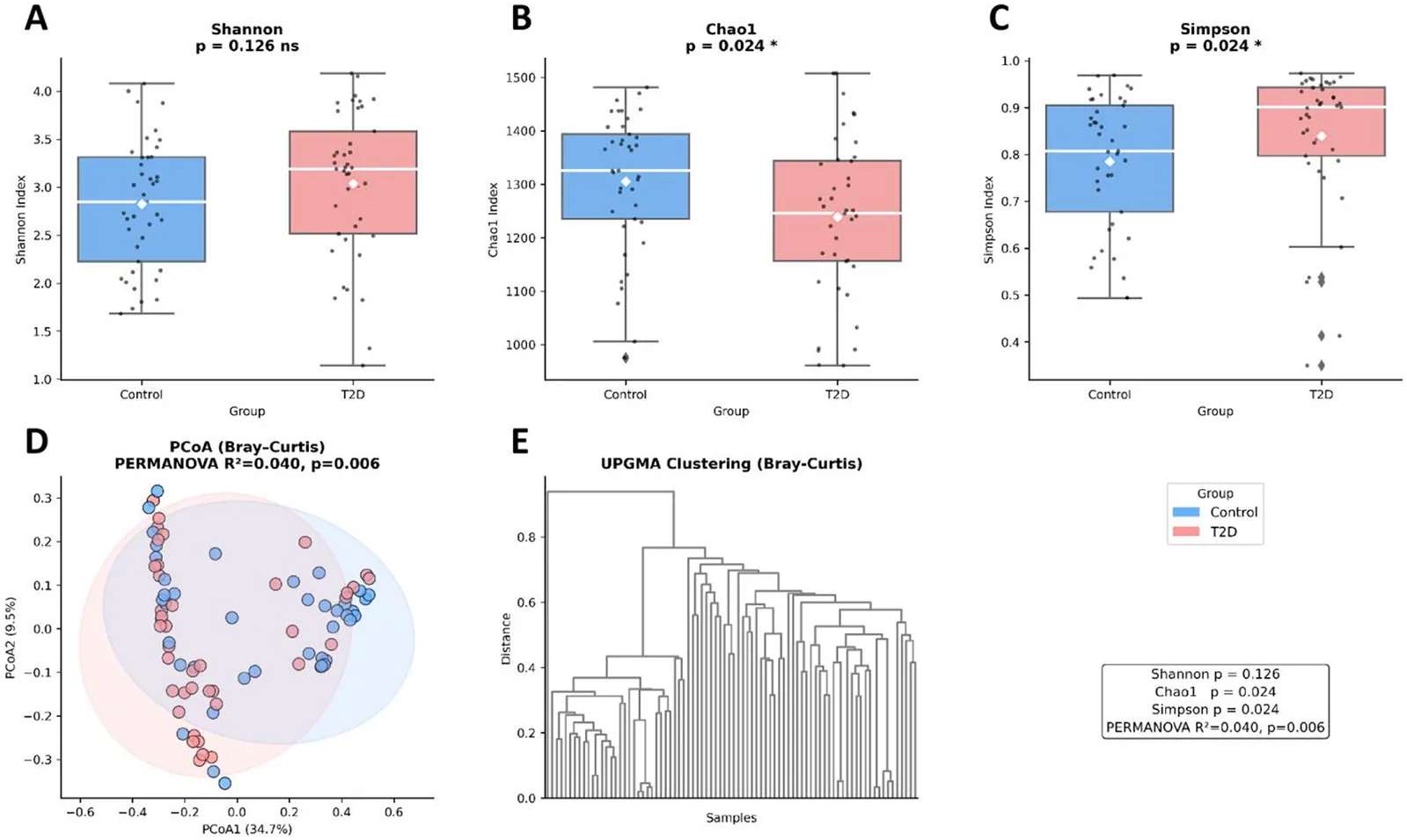
Alpha- and beta-diversity analyses of the gut microbiome in T2D and control subjects. **(A–C)** Comparison of alpha-diversity indices between groups, including Shannon diversity, Chao1 richness, and Simpson diversity. Boxes represent the interquartile range, center lines indicate medians, whiskers extend to 1.5 times the interquartile range, and individual points represent samples. Statistical significance was assessed using the two-sided Wilcoxon rank-sum test. Chao1 richness was significantly lower in the T2D group than in controls (*P* = 0.024), whereas Simpson diversity was significantly higher in T2D (*P* = 0.024). Shannon diversity did not differ significantly between groups (*P* = 0.126). **(D)** Principal coordinates analysis (PCoA) based on Bray-Curtis dissimilarity showing the overall compositional relationship between T2D and control samples. PERMANOVA indicated a significant but modest between-group difference in microbial community composition (R^2^ = 0.040, *P* = 0.006). **(E)** UPGMA hierarchical clustering dendrogram based on Bray-Curtis distance. ^*^P < 0.05; ns, not statistically significant.

Consistent with this pattern, beta-diversity analysis revealed a subtle but statistically significant between-group difference in overall taxonomic composition. Bray–Curtis-based principal coordinates analysis (PCoA) showed partial separation of T2D and control samples, and PERMANOVA confirmed a significant association between disease status and community composition (*R*^2^ = 0.040, *P* = 0.006), although the effect size was modest (Figure 5).

### 3.5 Exploratory functional trends do not survive multiple-testing correction

**Figure 6:**
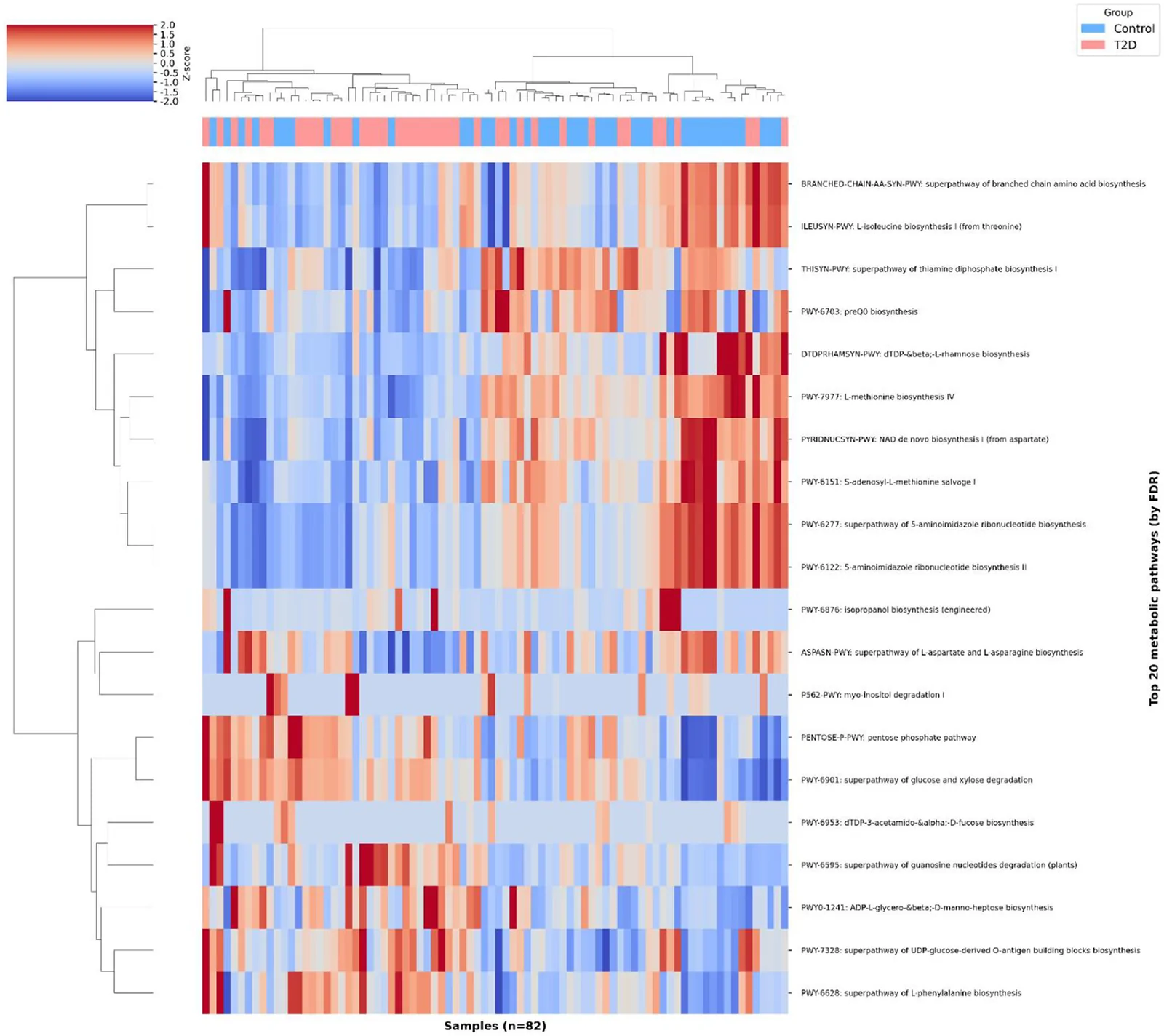
Exploratory pathway trends without FDR-significant differences. Heatmap of the top 20 microbial metabolic pathways ranked by Benjamini-Hochberg FDR-adjusted *P*-values from pathway-level differential abundance analysis. Although individual pathways did not consistently reach statistical significance after multiple testing correction, the analysis was used to explore coordinated functional trends across metabolic modules. These results should be interpreted as exploratory and hypothesis-generating rather than confirmatory.

To determine whether taxonomic alterations were accompanied by functional changes, we profiled microbial metabolic pathways from shotgun metagenomic data. A total of 22,961 pathways were detected, of which 486 passed abundance filtering. No individual pathway remained significant after Benjamini–Hochberg correction (Supplementary Figure 5, Supplementary Table 4). Consistent with this observation, hierarchical clustering of the top-ranked pathways revealed limited separation between T2D and control samples (Figure 6).

**Figure 7:**
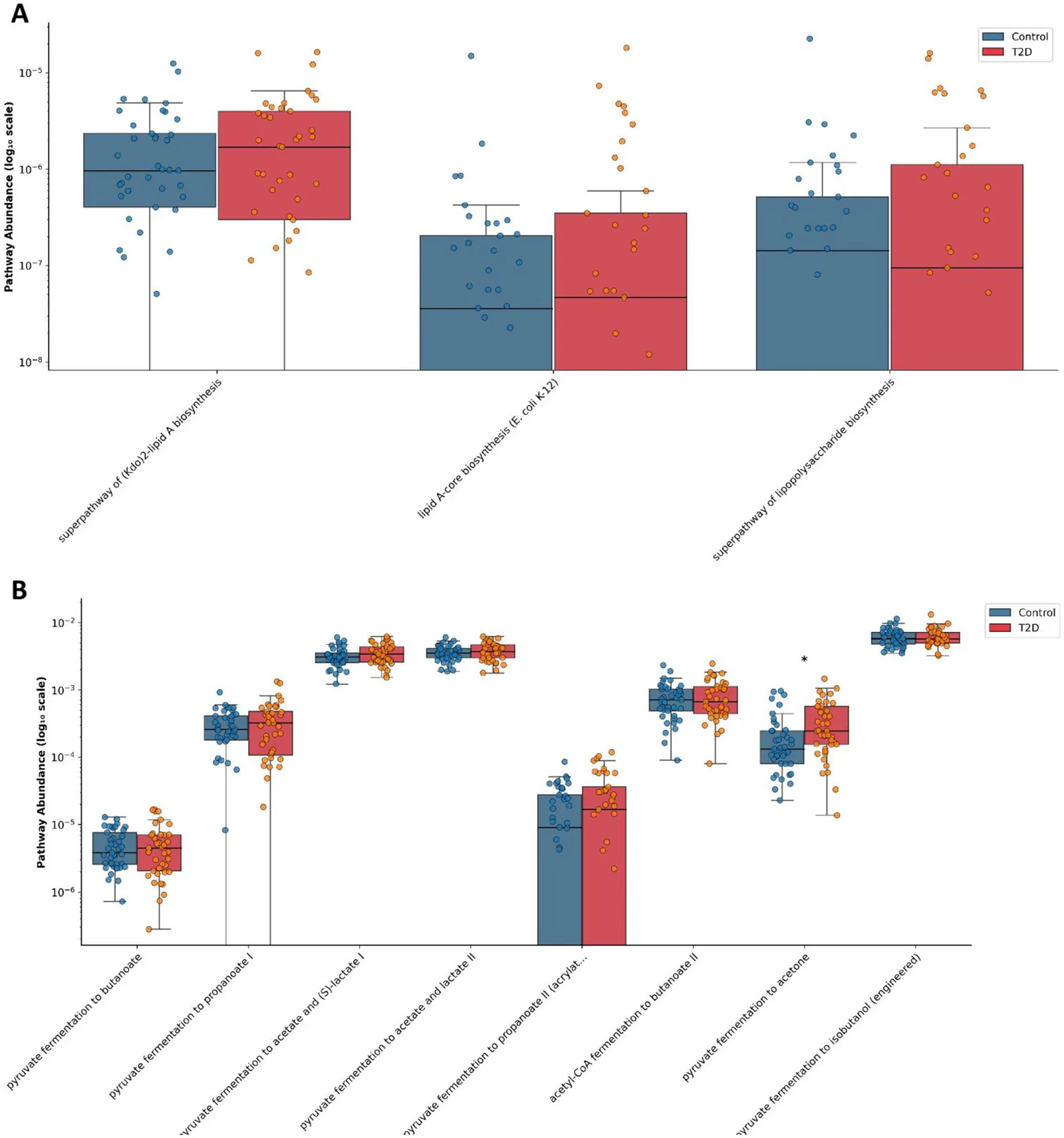
Selected metabolic pathways exhibit directional shifts in T2D. **(A)** Differential abundance of lipopolysaccharide biosynthesis pathways. **(B)** Differential abundance of short-chain fatty acid metabolism pathways. ^*^Indicates nominal unadjusted *P* < 0.05; no pathway remained significant after Benjamini-Hochberg FDR correction.

Despite weak univariate signals, coherent directional trends emerged across multiple biologically related functional modules. Pathways involved in lipopolysaccharide biosynthesis displayed higher abundance in T2D samples (Figure 7A). Similarly, pathways associated with short-chain fatty acid metabolism showed modest enrichment (Figure 7B). In contrast, several branched-chain amino acid biosynthesis pathways, including L-isoleucine biosynthesis I, L-isoleucine biosynthesis III, L-valine biosynthesis, and the superpathway of branched-chain amino acid biosynthesis, exhibited consistently higher abundance in controls (Supplementary Figure 6). Subtle but coordinated shifts were also observed in bile acid metabolism-related pathways (Supplementary Figure 7; Supplementary Table 5).

Although several related metabolic modules showed directional trends, no pathway survived multiple-testing correction. These observations are therefore descriptive and hypothesis-generating; they do not demonstrate coordinated functional remodeling and require validation in larger, medication-resolved, and functionally measured datasets.

### 3.6 Exploratory host–microbiome associations with glycemic status

**Figure 8:**
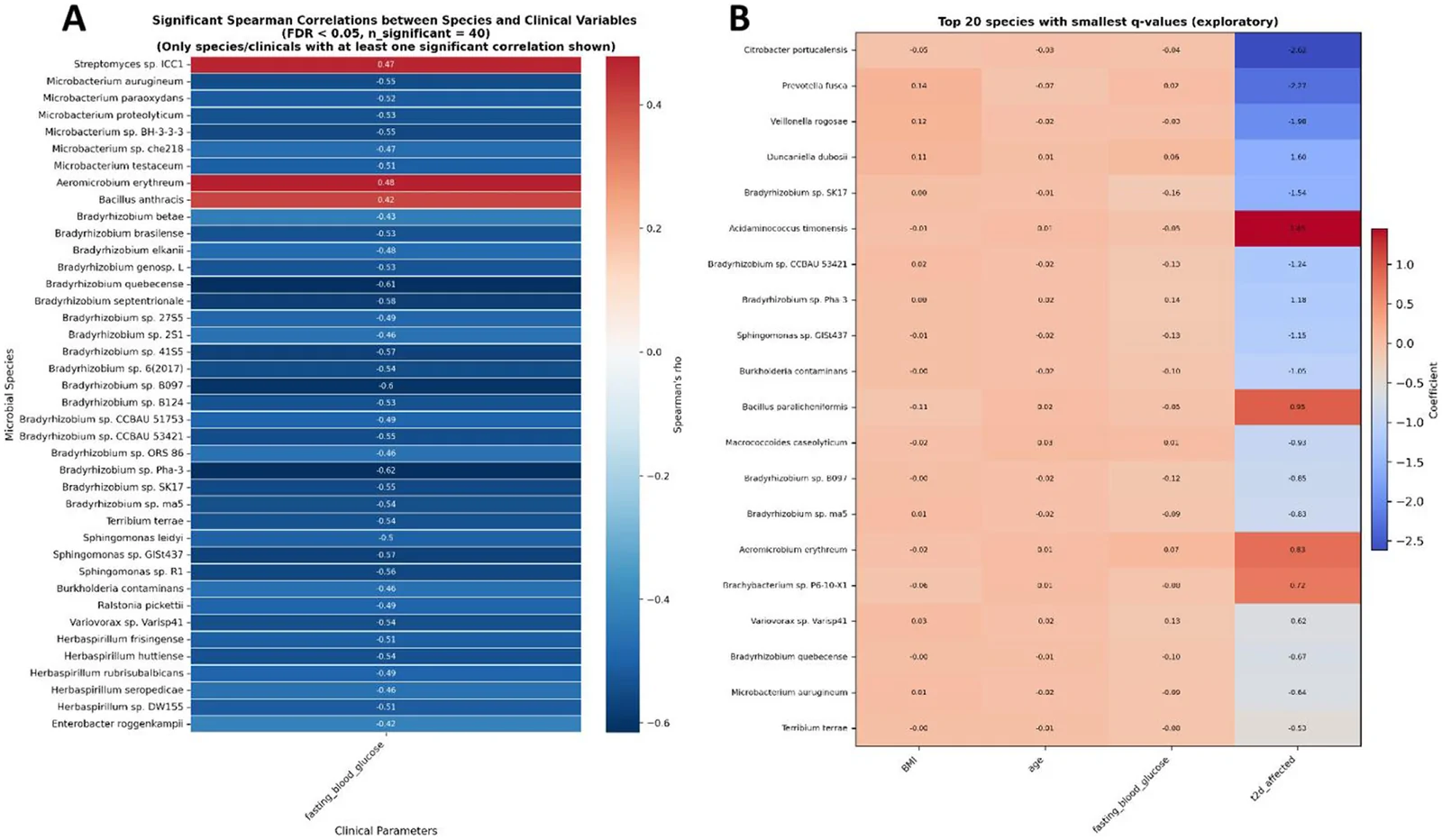
Exploratory associations between fasting blood glucose and microbial features. **(A)** Heatmap of significant Spearman correlations between microbial species and host clinical variables. **(B)** Multivariable association analysis adjusted for age, BMI, fasting blood glucose, and T2D status.

We examined exploratory associations between microbial features and clinical variables. Spearman analysis identified 40 FDR-adjusted associations involving species-level assignments and host phenotypes (Supplementary Table 6). The numerically strongest correlations with fasting blood glucose involved *Bradyrhizobium* assignments, including *Bradyrhizobium* sp. Pha-3 (ρ = −0.616), Bradyrhizobium quebecense (ρ = −0.609), and *Bradyrhizobium* sp. B097 (ρ = −0.603; Figure 8A). Because these are low-abundance environmental taxa and the input data are compositional, these coefficients may reflect contamination, taxonomic misassignment, batch structure, or induced correlation rather than biological coupling.

Multivariable regression adjusting for age, BMI, fasting blood glucose, and T2D status identified 10 associations after multiple-testing correction (FDR < 0.05) (Figure 8B; Supplementary Table 7). Fasting blood glucose and disease status accounted for the largest modeled associations in the available covariate set. Partial-effect analyses retained a negative statistical association between fasting blood glucose and *Bradyrhizobium* assignments and a positive association between T2D status and *Escherichia coli* abundance (Supplementary Figure 8). Neither finding establishes a disease-driven effect: the *Bradyrhizobium* signal is technically uncertain, and *E. coli* enrichment has been associated with metformin exposure, for which complete data were unavailable in this cohort (Forslund et al., 2015).

In contrast, pathway-level analyses revealed substantially weaker host–microbiome coupling. Correlation coefficients were generally modest (|ρ| ≤ 0.43), and no pathway-level associations remained significant following multiple-testing correction (Supplementary Figures 9, 10; Supplementary Tables 8, 9).

Within the measured covariates, glycemic status showed the strongest statistical association with microbial variation. This within-cohort pattern remains exploratory and may be affected by treatment, compositionality, unmeasured confounding, and technically uncertain taxa; it should not be interpreted as evidence that glycemia is the principal biological driver of the microbiome.

### 3.7 Conserved enterotype states exhibit disease-associated occupancy shifts

**Figure 9:**
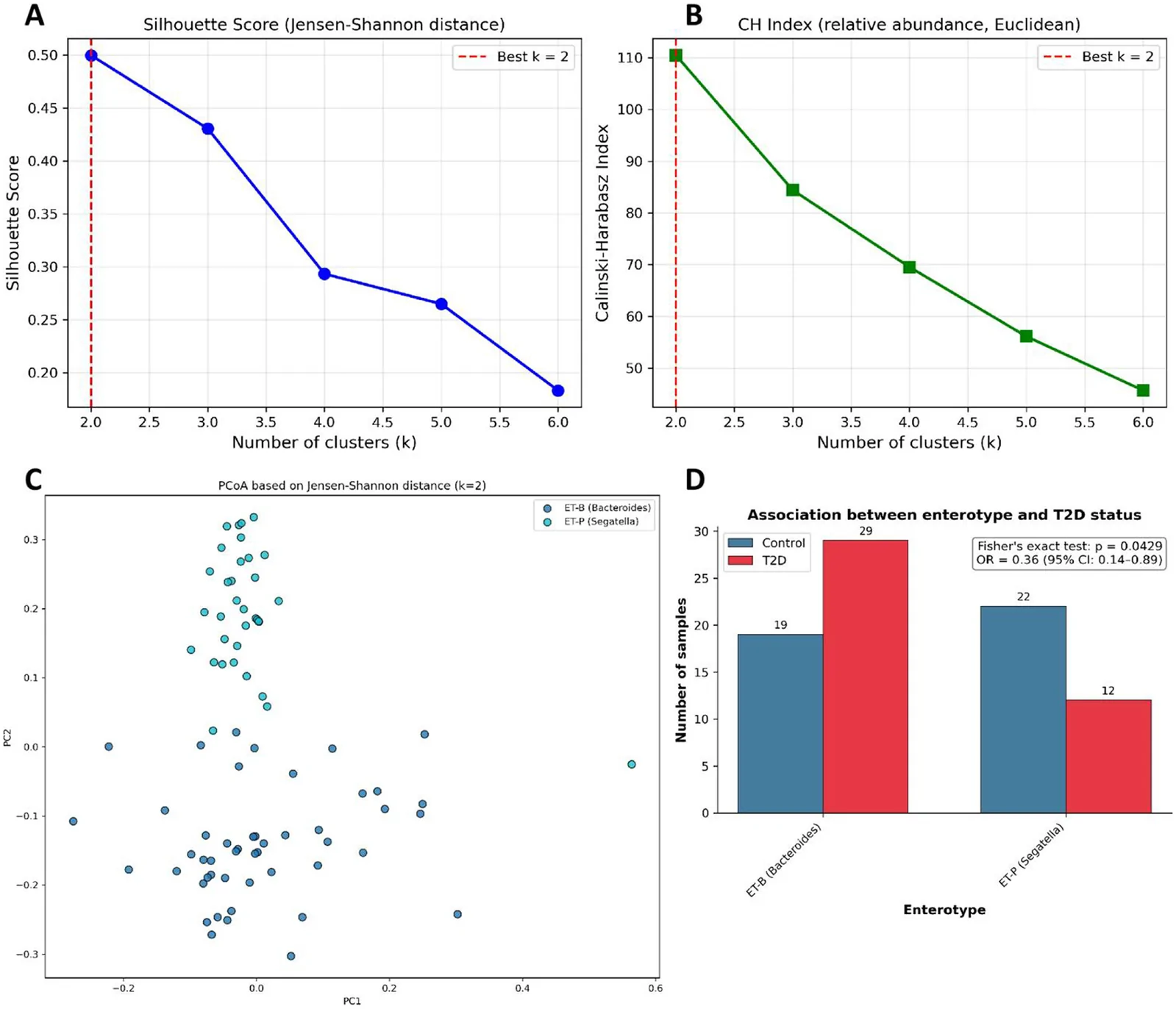
Conserved enterotype structure displays disease-associated occupancy shifts. **(A)** Silhouette scores from Partitioning Around Medoids clustering based on Jensen-Shannon distance across candidate cluster numbers; the highest score was observed at *k* = 2. **(B)** Calinski-Harabasz index based on Euclidean distances between genus-level relative-abundance profiles across candidate cluster numbers; the highest value was observed at *k* = 2. **(C)** Principal coordinates analysis based on Jensen-Shannon distance showing separation between the Bacteroides-dominated enterotype (ET-B) and the Segatella-dominated enterotype (ET-P). **(D)** Distribution of the two enterotypes across control and T2D groups. Differences in enterotype prevalence were evaluated using Fisher's exact test (*P* = 0.0429; OR = 0.36, 95% CI: 0.14–0.89).

To evaluate large-scale community organization, enterotype analysis was performed using Jensen–Shannon distance and partitioning around medoids. Two robust enterotypes were identified (*k* = 2), supported by silhouette and Calinski–Harabasz statistics (Figures 9A, B). The first enterotype (ET-B; *n* = 48) was dominated by Bacteroides, whereas the second (ET-P; *n* = 34) was characterized by Segatella dominance (Figure 9C).

Although both enterotypes were observed in both clinical groups, their relative distributions differed significantly according to disease status (Fisher's exact test, *P* = 0.0429). The Segatella-dominated enterotype was associated with reduced odds of T2D (OR = 0.36, 95% CI: 0.14–0.89), whereas the Bacteroides-dominated enterotype was more prevalent among diabetic individuals (Figure 9D; Supplementary Table 10).

These observations indicate that T2D does not generate novel community configurations. Instead, disease influences occupancy within pre-existing ecological states, resulting in altered enterotype distributions while preserving overall enterotype architecture.

### 3.8 Spearman-based co-occurrence networks differ in topology between groups

**Figure 10:**
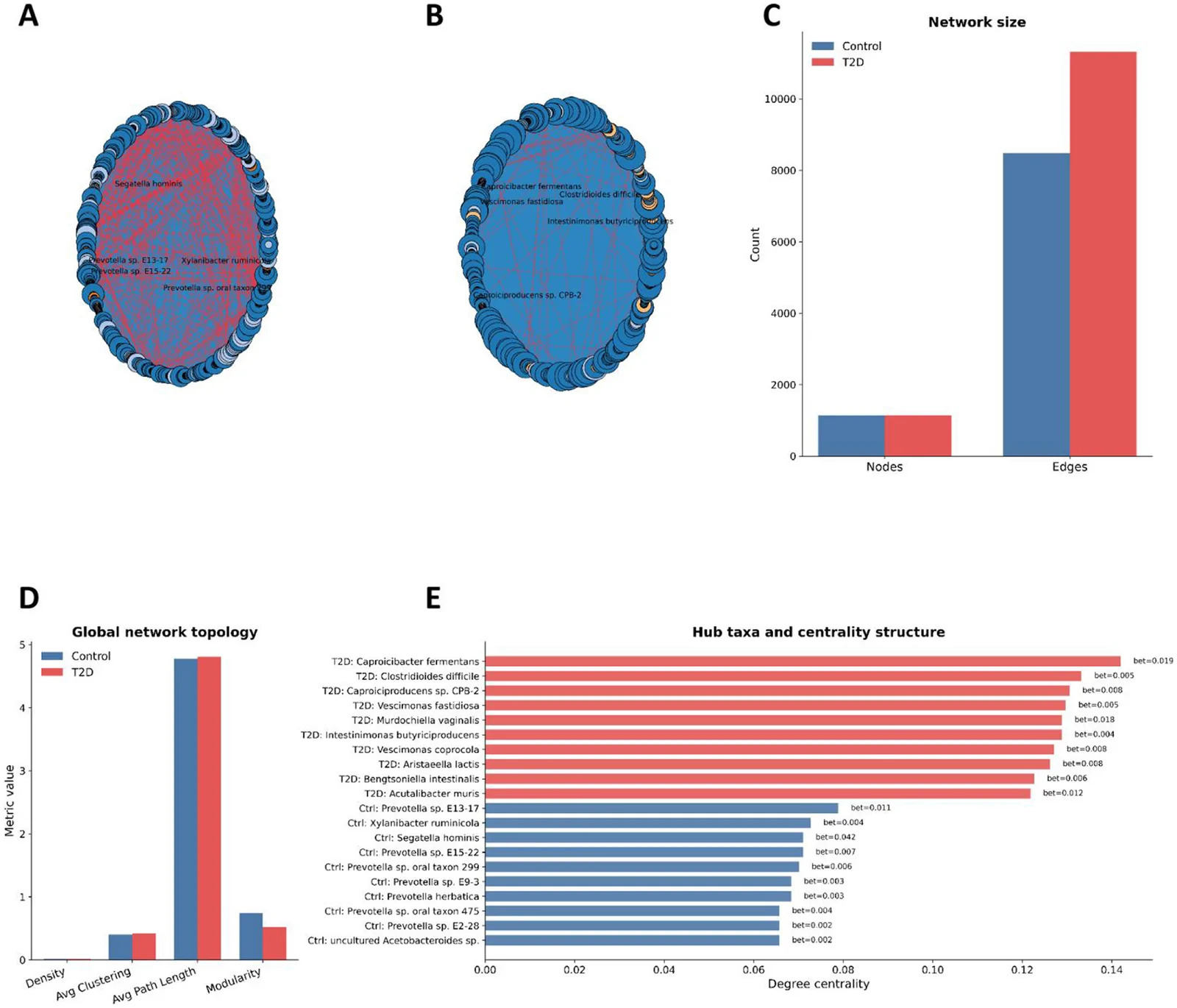
Spearman-based co-occurrence networks differ in topology between T2D and control groups. **(A)** Species co-occurrence network constructed from control samples. **(B)** Species co-occurrence network constructed from T2D samples. **(C)** Comparison of basic network size between groups, including node and edge counts. **(D)** Comparison of global and mesoscopic network topology between groups, including network density, average degree, average clustering coefficient, average path length, modularity, and number of detected modules. **(E)** Top hub taxa ranked by degree centrality in control and T2D networks, with betweenness centrality annotated for each taxon. Nodes represent microbial species, and node size is proportional to degree centrality. Node colors indicate module membership identified by Louvain community detection. Edges represent significant pairwise co-occurrence relationships defined by Spearman correlation (|ρ| ≥ 0.6, FDR < 0.05). Under the selected Spearman threshold, the T2D graph had higher edge density and lower modularity than the control graph. Because relative-abundance data are compositional and sparse, and no compositionality-aware estimator was used, these topology differences may include induced correlations and threshold instability. They are descriptive, pipeline-dependent statistical co-occurrence patterns and should not be interpreted as increased biological connectivity, ecological rewiring, or mechanistic interaction.

We constructed exploratory species-level Spearman co-occurrence networks separately for control and T2D samples. Both thresholded graphs contained the same number of retained species (1,142 nodes each), whereas their edge sets and summary topology differed (Figures 10A–D). Given the compositional input, sparsity, modest group size, and use of a fixed correlation threshold, these graphs summarize statistical covariance under the selected pipeline and do not establish microbial interaction architecture.

Under the selected Spearman threshold, the T2D graph had more edges (11,314 vs. 8,477), higher density (0.0174 vs. 0.0130), higher average degree (19.81 vs. 14.85), and a modestly higher average clustering coefficient (0.418 vs. 0.400), whereas average path length was similar (4.81 vs. 4.78). These are descriptive properties of the inferred graphs; they do not demonstrate increased biological connectivity.

Louvain analysis yielded lower modularity in the T2D graph than in the control graph (0.521 vs. 0.740), with a similar number of detected modules (219 vs. 224). Statistical hub rankings also differed, with control hubs predominantly assigned to Prevotellaceae-related species and T2D hubs enriched for Bacillota-associated species (Figure 10D). Because modularity and centrality inherit uncertainty from the correlation matrix and edge threshold, these contrasts are hypothesis-generating and require replication with compositionality-aware inference and independent cohorts.

Together, these analyses identify between-group differences in Spearman-based co-occurrence summaries. They do not establish ecological network rewiring, increased biological connectivity, or altered microbial interactions, and should be tested using compositionally robust methods and external data.

### 3.9 Within-cohort machine-learning models identify exploratory signatures

**Figure 11:**
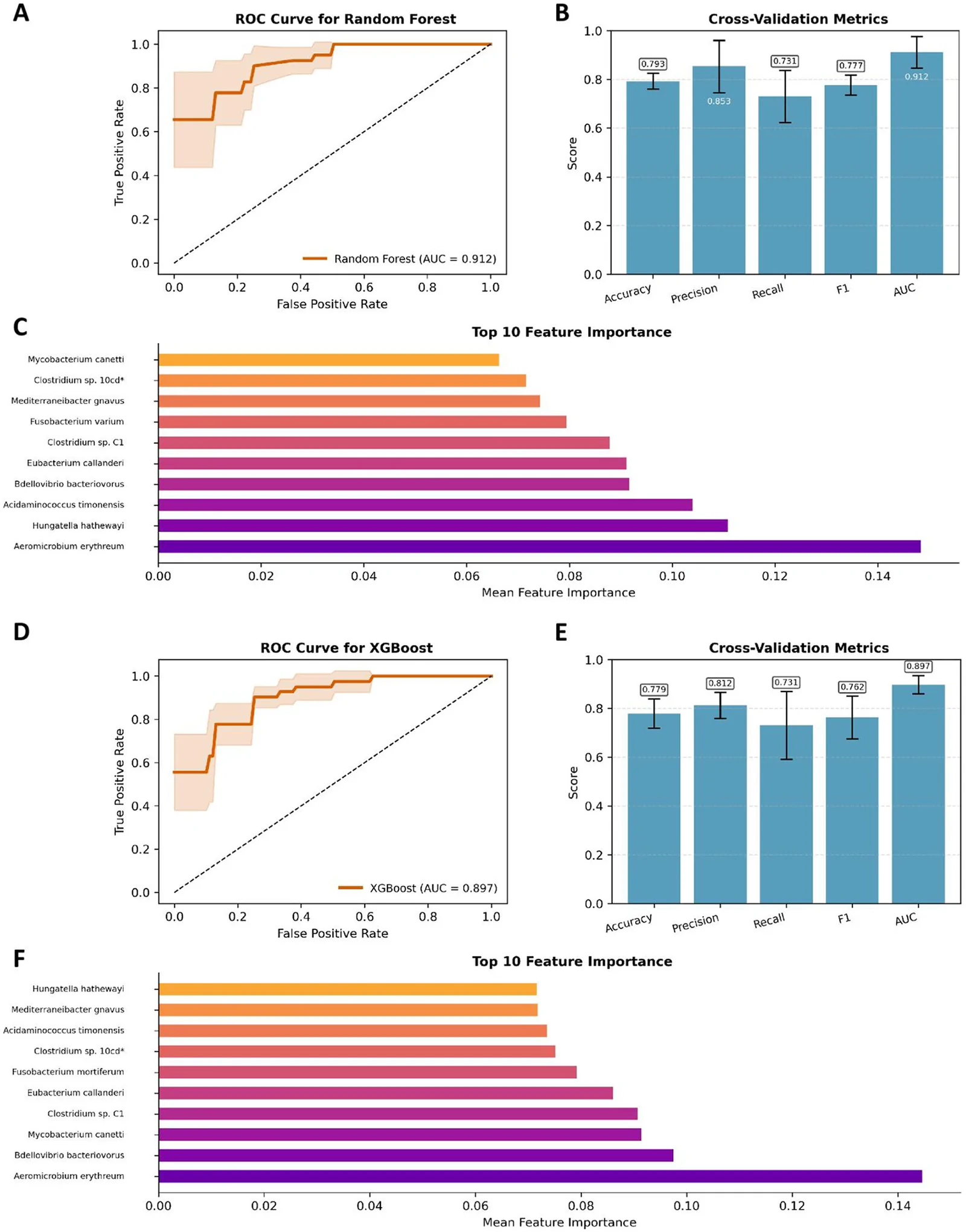
Within-cohort machine-learning models identify exploratory T2D-associated signatures. **(A)** Receiver operating characteristic (ROC) curve for Random Forest classification. **(B)** Cross-validation performance metrics for Random Forest. **(C)** Feature importance ranking for Random Forest. **(D)** ROC curve for XGBoost classification. **(E)** Cross-validation performance metrics for XGBoost. **(F)** Feature importance ranking for XGBoost. Model performance was evaluated using stratified five-fold internal cross-validation. Reported metrics include ROC-AUC, accuracy, precision, recall, and F1-score. Feature selection and preprocessing were restricted to training folds. Because the cohort comprised 82 participants and no independent external validation was performed, the estimates describe within-cohort discrimination and not clinical diagnostic performance or generalizability.

To evaluate within-cohort discriminative information, we applied two machine-learning approaches. In stratified five-fold internal cross-validation, species-based Random Forest and XGBoost models yielded AUC values of 0.912 and 0.897, respectively (Figures 11A–F), whereas pathway-based models yielded AUC values of approximately 0.75–0.77 (Supplementary Figure 11). With only 82 participants and no independent test cohort, these estimates are susceptible to overfitting and population-specific structure and should not be interpreted as externally validated diagnostic performance.

**Figure 12:**
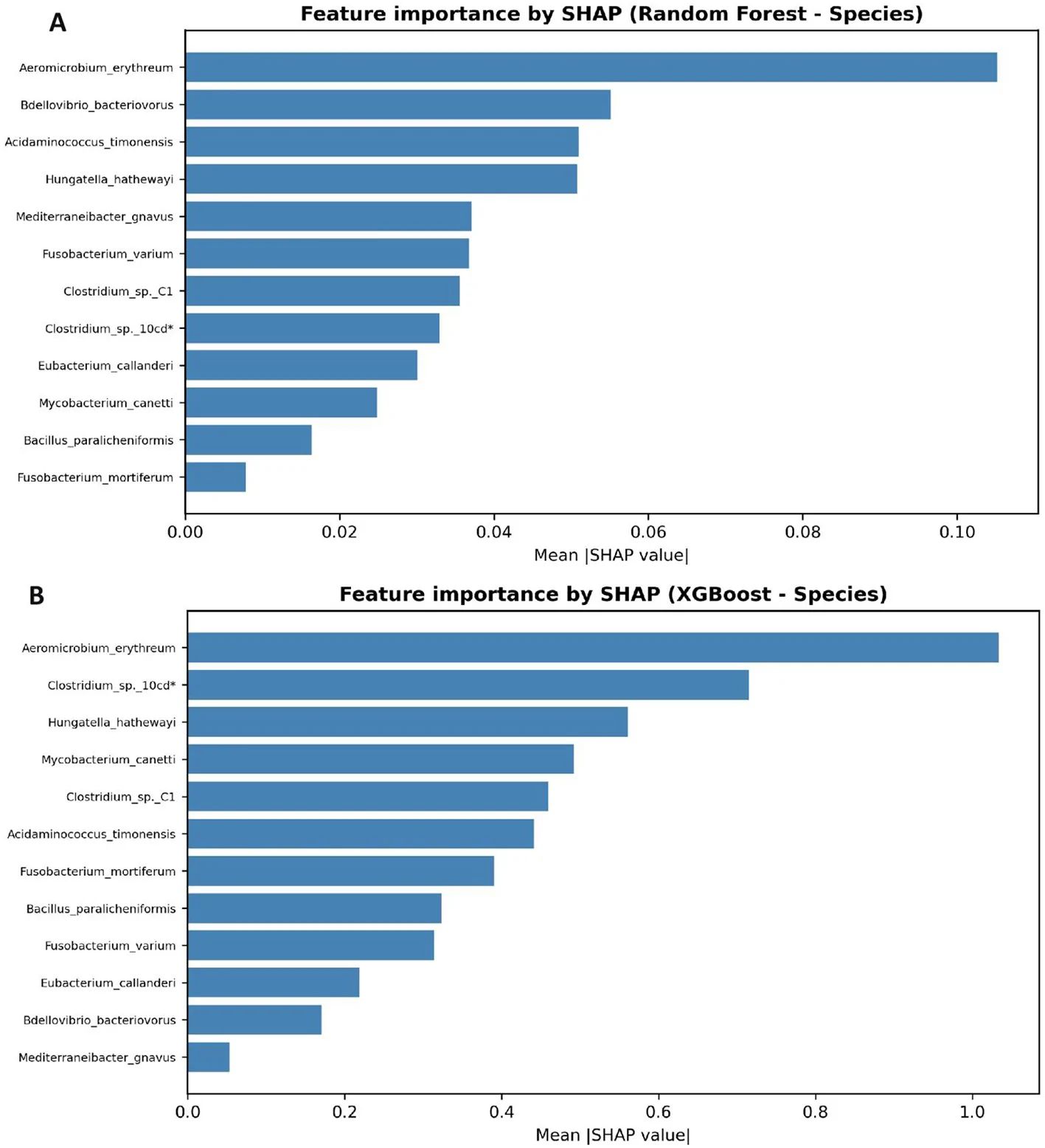
Within-cohort SHAP summaries of feature contributions across models. **(A)** Feature importance based on SHAP values for Random Forest. **(B)** Feature importance based on SHAP values for XGBoost. SHAP analysis was used to quantify feature contributions to model predictions in an additive and interpretable manner, enabling both global and local explanation of microbial signatures associated with T2D.

Several taxa and pathways contributed to classification within this dataset (Supplementary Figure 12), including Aeromicrobium erythreum and pathways involved in chitin degradation and O-antigen biosynthesis (Supplementary Table 11). SHAP rankings showed partial overlap across the two algorithms (Figures 12A, B); the corresponding summary plots are shown in Supplementary Figures 13, 14. This within-dataset overlap is not independent validation, and the environmental-taxon features remain technically uncertain.

Feature overlap across the two algorithms indicates within-dataset consistency, but it does not establish robustness across populations. In particular, Aeromicrobium erythreum and other low-abundance environmental assignments have not emerged as established T2D markers in the Qin or Karlsson cohorts (Qin et al., 2012; Karlsson et al., 2013) and may be pipeline- or cohort-specific. Direct model transfer was not undertaken because the legacy cohorts used different reference spaces, feature definitions, population structures, and treatment metadata; independent reprocessing from raw reads and harmonized medication data will be required for a valid external test.

### 3.10 Multi-layer integration of microbiome alterations in T2D

Across taxonomic, functional, enterotype, Spearman-based co-occurrence, and machine-learning analyses, the cohort showed preserved higher-order community structure together with finer-scale statistical differences. Because medication data were incomplete, environmental-taxon signals were technically uncertain, correlation analyses were not compositionality-aware, and machine-learning models lacked external validation, these layers should be interpreted as complementary exploratory descriptions rather than evidence of coordinated ecological rewiring or generalizable predictive signatures.

## 4 Discussion

### 4.1 Principal findings: a multi-layer characterization of microbiome variation in T2D

In this study, we integrated shotgun metagenomic profiling with enterotype analysis, ecological network inference, and interpretable machine-learning approaches to characterize gut microbiome variation associated with type 2 diabetes (T2D) in a Chinese Han cohort. Rather than focusing exclusively on differential abundance of individual taxa, we examined microbiome variation across multiple organizational levels, including taxonomic composition, functional potential, community-state organization, microbial interaction architecture, and discriminative microbial signatures.

Across these complementary analytical layers, we observed a consistent pattern in which higher-order community organization remained broadly preserved, whereas finer-scale analyses revealed selective differences in species composition, community diversity, functional profiles, ecological network structure, and microbial signatures identified by machine-learning models. Collectively, these observations are consistent with a systems-level interpretation of microbiome variation in T2D and remain broadly consistent with previous reports describing distributed microbial and functional alterations in metabolic disease. Because the present study is cross-sectional, these findings should be interpreted as associative ecological observations rather than evidence of causal microbiome remodeling or a fundamentally new disease mechanism. Instead, our findings complement existing taxonomic and functional observations by integrating multiple analytical perspectives within the same cohort.

A systemic pathophysiological interpretation must connect ecological observations to host-facing mechanisms while preserving the direction of evidence. Microbial metabolites may influence glucose homeostasis through short-chain-fatty-acid signaling, bile-acid transformation, branched-chain-amino-acid metabolism, and enteroendocrine pathways; barrier impairment and lipopolysaccharide exposure may promote innate immune activation and chronic low-grade inflammation (Cani et al., 2007; Gurung et al., 2020; Canfora et al., 2019; Morrison and Preston, 2016; Wahlstrom et al., 2016; Cani and Jordan, 2018). At the same time, hyperglycemia, diet, adiposity, oral inflammation, and pharmacotherapy can reshape microbial habitats. The oral and intestinal microbiota may therefore represent linked but distinct compartments of a broader host microecological system (Guo et al., 2023). Our fecal cross-sectional data do not test this oral–gut axis or establish any of these mechanisms, but they motivate future multi-site, metabolomic, immunologic, and longitudinal studies.

A central interpretive constraint is the lack of complete antidiabetic medication data. Metformin has a well-established microbiome signature, including enrichment of Escherichia and changes in functional pathways that overlap with T2D-associated signals (Forslund et al., 2015). Other glucose-lowering therapies may also alter the intestinal environment. Therefore, the taxa, functional trends, co-occurrence patterns, and within-cohort classifier signals observed here cannot be assigned specifically to untreated T2D pathophysiology; throughout this Discussion they are described as associations potentially combining disease, treatment, and other host effects.

### 4.2 Preserved higher-order structure with fine-scale taxonomic divergence

At higher taxonomic levels, gut microbial community composition remained broadly stable between T2D and healthy individuals. Dominant phyla and overall community-state structure were largely preserved, consistent with previous reports suggesting that broad microbiome architecture may remain relatively resilient in metabolic disease contexts [Qin et al., 2012; Karlsson et al., 2013; The Integrative HMP (iHMP) Research Network Consortium, 2019]. In contrast, increasing taxonomic resolution revealed progressively stronger divergence. Species-level analyses identified a subset of taxa showing reproducible abundance differences between groups, whereas genus-level differences were comparatively limited. This hierarchical pattern suggests that disease-associated variation may emerge more readily at finer taxonomic resolution than at the level of dominant higher-order community structure.

From an ecological perspective, such a pattern is compatible with the possibility that altered host metabolic conditions reshape selective pressures acting on microbial communities without necessarily replacing the dominant community scaffold (Prosser et al., 2007; Flores et al., 2025; Sabih Ur Rehman et al., 2025; Widder et al., 2016). Rather than implying deterministic effects of a few individual taxa, the observed structure is more consistent with distributed ecological filtering across multiple community members. This interpretation aligns with prior metagenomic studies of T2D that have reported broad microbial and functional alterations rather than complete ecosystem disruption. Qin et al. (2012) first showed that T2D is associated with changes in microbial gene content and functional capacity, whereas Karlsson et al. (2013) identified disease-associated microbial signatures in European women with impaired glucose regulation. Subsequent work by Forslund et al. (2015) demonstrated that treatment-related confounding, particularly metformin exposure, can substantially reshape apparent T2D-associated microbiome profiles. Taken together, these previous studies and our findings support an interpretation of distributed, treatment-confounded microbiome variation without evidence of complete ecosystem disruption; they do not establish coordinated ecological restructuring.

### 4.3 Exploratory functional trends and mechanistic context

Functional profiling revealed that individual metabolic pathways rarely remained significant after correction for multiple testing. Nevertheless, pathways belonging to related biochemical categories exhibited coordinated directional trends across samples, including lipopolysaccharide biosynthesis, short-chain fatty acid metabolism, branched-chain amino acid metabolism, and bile acid-related functions. This pattern suggests that functional alterations in T2D may be distributed across metabolic modules rather than concentrated in a small number of individually dominant pathways. Such a scenario is compatible with the concept of functional redundancy in microbial ecosystems, in which multiple taxa contribute overlapping metabolic capacities and thereby buffer ecosystem-level function despite taxonomic turnover (Louca et al., 2018; Diener et al., 2020; Fan and Pedersen, 2021; Li et al., 2023; Seppi et al., 2024; Kadibalban et al., 2025; Moya and Ferrer, 2016). Although these pathway-level signals should be interpreted cautiously, their directional consistency is notable because the implicated functional categories overlap with mechanisms previously linked to T2D pathophysiology, including intestinal barrier regulation, metabolic endotoxemia, host inflammatory tone, and bile acid-mediated metabolic signaling (Cani et al., 2007; Canfora et al., 2019; Morrison and Preston, 2016; Wahlstrom et al., 2016; Cani and Jordan, 2018). We therefore view these findings not as direct mechanistic evidence, but as hypothesis-generating ecological signals suggesting that coordinated functional variation may accompany T2D-associated microbiome variation. Further validation using metabolomic, transcriptomic, and longitudinal data will be necessary to determine whether these pathway trends correspond to reproducible biological alterations at the host–microbiome interface.

### 4.4 Glycemic status as an ecological correlate of microbiome variation

Among host clinical variables, fasting blood glucose showed the strongest and most consistent association with microbial variation in the present cohort. Species-level associations with glycemic status were more evident than pathway-level associations, suggesting that host–microbiome relationships in T2D may be distributed across multiple ecological components rather than mediated through a single dominant functional axis. These observations are compatible with the possibility that glycemic status acts as an important ecological correlate of gut microbial variation, potentially through effects on intestinal physiology, nutrient availability, immune tone, and the intestinal barrier environment (Fan and Pedersen, 2021). More broadly, they are consistent with current models linking hyperglycemia, microbial metabolites, low-grade inflammation, and host metabolic dysfunction within the pathophysiology of T2D.

At the same time, the apparent strength of an association does not rescue an ecologically implausible feature from technical uncertainty. *Bradyrhizobium* assignments were retained in univariate and multivariable analyses, yet members of this genus are predominantly environmental organisms and the study included no extraction blanks or orthogonal confirmation. A contaminant or misclassified sequence can correlate strongly with fasting glucose if reagent lot, extraction batch, sequencing run, or another unrecorded technical factor is aligned with clinical group; compositional closure can further propagate such structure across relative abundances (Gloor et al., 2017; Kurtz et al., 2015; Salter et al., 2014). We therefore do not interpret *Bradyrhizobium* as a core gut taxon, protective organism, or mechanistic contributor. Instead, its high statistical contribution is a warning that batch effects or experimental artifacts may influence the feature space. Overall, the data identify exploratory associations with glycemic status but do not establish directionality, causality, or biological validity of the environmental-taxon signals.

### 4.5 Enterotype stability with altered ecological occupancy

Enterotype analysis identified two major community configurations shared across T2D and control individuals. Rather than indicating the emergence of a novel diabetes-specific community state, T2D was associated primarily with altered occupancy of pre-existing enterotype configurations. This observation is compatible with ecological models in which host-associated perturbations modify the relative probability of occupying alternative community states without necessarily generating entirely new configurations (Flores et al., 2025; Mendes-Soares and Chia, 2017).

However, the biological interpretation of enterotypes remains debated (Costea et al., 2018). Several studies have argued that gut microbiome variation may be better represented as continuous ecological gradients rather than as strictly discrete categories. Accordingly, our findings should not be interpreted as evidence for rigid enterotype classes, but rather as support for structured community-state variation in which T2D may shift ecological occupancy within an already existing microbial landscape.

### 4.6 Limitations of Spearman-based co-occurrence topology

The Spearman-based graphs differed in their summary topology despite relatively modest shifts in overall taxonomic composition. The T2D graph retained the same number of nodes as the control graph but had more thresholded edges, higher density and average degree, modestly higher clustering, and lower modularity. These values describe covariance patterns produced by the selected estimator and threshold. Given the compositional and sparse data, they cannot determine whether the underlying microbiome was more interconnected or less compartmentalized in ecological terms.

These topology differences are hypothesis-generating. Relative-abundance data are constrained to a constant sum, so an increase in one component necessarily changes the proportions of others; sparsity and zero inflation further destabilize pairwise ranks. Conventional Spearman correlations can therefore create, suppress, or reverse edges, and thresholding magnifies these effects in density, degree, modularity, and hub rankings (Gloor et al., 2017; Kurtz et al., 2015). Shared host conditions, medication exposure, diet, or technical batches may also produce covariance without ecological interaction (Faust and Raes, 2012; Philippot et al., 2021; Esquivel-Hernandez et al., 2023; Xu et al., 2025; Rottjers and Faust, 2018; Corral Lopez et al., 2026). Compositionally aware frameworks such as SPIEC-EASI, SparCC, or FlashWeave, together with stability analysis and external replication, would provide a more defensible test. We therefore interpret Figure 10 only as a descriptive sensitivity analysis of statistical co-occurrence under the present pipeline, not as evidence of increased connectivity, ecological rewiring, or direct interactions in T2D.

### 4.7 Within-cohort machine-learning signatures and generalizability

The Random Forest and XGBoost models captured discriminative information within this cohort, and partial overlap of ranked features indicates algorithmic consistency on the same data. However, agreement between algorithms is not independent biological validation. With 41 cases and 41 controls, five-fold cross-validation leaves small validation folds and cannot measure transportability to different populations, sequencing platforms, taxonomic databases, or treatment distributions (Papoutsoglou et al., 2023; Probul et al., 2024). The leading low-abundance environmental assignments further increase the possibility of cohort- or batch-specific fitting. The Qin and Karlsson studies identified T2D-associated microbial patterns in different populations (Qin et al., 2012; Karlsson et al., 2013), but their legacy feature spaces and profiling strategies are not directly interchangeable with the GTDB R207 species assignments used here; notably, our leading *Aeromicrobium* feature is not an established cross-cohort T2D marker. A valid external evaluation would require harmonized raw-read reprocessing, compatible clinical definitions, and medication-stratified analysis. Accordingly, the reported AUCs are exploratory within-cohort estimates, and the models are neither clinically validated nor demonstrated to generalize.

### 4.8 An integrative but provisional interpretation of T2D-associated microbiome variation

Taken together, the analyses offer an integrated description of gut microbiome variation in T2D that extends beyond a single taxon list. The observed patterns do not indicate ecosystem collapse or a discrete disease-specific community state. They show modest differences across several analytical layers, but the apparent concordance may combine disease status with treatment, other host exposures, compositional effects, and technical structure. We therefore use the systems-level framework as an organizing hypothesis rather than evidence of coordinated ecological perturbation, altered interactions, or deterministic microbial drivers.

This interpretation remains provisional and does not exclude alternative explanations, including continuous compositional gradients, cohort-specific ecological effects, or residual confounding from incompletely measured host and treatment variables. Nevertheless, it provides a useful conceptual basis for future studies aimed at integrating metagenomics with detailed host phenotyping, metabolomics, and longitudinal sampling to define ecosystem-level microbial signatures with clearer mechanistic and translational relevance.

### 4.9 Limitations and future directions

Several limitations should be considered when interpreting the present findings. First, the cross-sectional design precludes inference of temporal relationships and causality; longitudinal studies will be required to determine whether the observed microbial patterns precede, accompany, or follow metabolic alterations. Second, although deep shotgun metagenomic sequencing provides broad taxonomic and functional resolution, these data primarily capture functional potential rather than actual microbial activity. Integration with transcriptomic, proteomic, and metabolomic datasets would therefore improve mechanistic resolution.

Third, the relative-abundance data are compositional, sparse, and zero-inflated. Rank-based differential tests and Spearman correlations do not fully address these properties and may yield induced associations (Gloor et al., 2017; Kurtz et al., 2015). This limitation is especially consequential for Figure 10: spurious or unstable pairwise correlations are converted into edges, so network density, average degree, modularity, and hub rankings may change even in the absence of altered ecological interactions. The network results must therefore be viewed as exploratory summaries specific to the present estimator and threshold. Future work should compare ANCOM-BC or ALDEx2 for differential abundance and compositionally robust network approaches such as SPIEC-EASI, SparCC, or FlashWeave, with resampling-based stability assessment and independent replication. Pathway-level trends also remain exploratory because no pathway survived multiple-testing correction.

Fourth, antidiabetic medication data were not uniformly available and this is a major limitation of the study. Metformin and other glucose-lowering therapies can reshape gut community composition and metabolic potential; metformin-associated enrichment of Escherichia and shifts in short-chain-fatty-acid-related functions can be mistaken for disease signatures (Forslund et al., 2015). Consequently, the observed *E. coli* enrichment, other positive taxa, functional trends, network differences, and classifier performance cannot be attributed specifically to T2D pathophysiology. Diet, physical activity, and other lifestyle variables were also incompletely measured. Residual treatment and lifestyle confounding may therefore account for part of the reported multi-layer pattern, and no causal or disease-specific interpretation is warranted.

Finally, the study included only 41 cases and 41 controls from a single regional Chinese cohort. Internal cross-validation reduces leakage but cannot exclude overfitting to population-, batch-, or treatment-specific characteristics. No independent cohort was used. Direct transfer to the Qin and Karlsson datasets is constrained by non-harmonized raw-read processing, taxonomic reference spaces, population structure, and medication metadata (Qin et al., 2012; Karlsson et al., 2013), and a *post hoc* comparison of non-equivalent features would risk misleading inference. Larger external cohorts should be reprocessed through a common pipeline and analyzed with medication-stratified models before any claim of diagnostic generalizability. The present machine-learning results are therefore exploratory within-cohort estimates only.

## 5 Conclusion

In conclusion, this study provides a multi-layer metagenomic characterization of gut microbiome variation associated with type 2 diabetes (T2D) in a Chinese Han cohort. Higher-order community structure was broadly preserved, whereas exploratory differences were observed in species composition, functional trends, community-state occupancy, Spearman-based co-occurrence summaries, and within-cohort machine-learning signatures.

Rather than indicating a discrete disease-specific microbiome or ecosystem collapse, the results are consistent with distributed variation across multiple analytical levels within a largely conserved microbial framework; treatment, compositional, technical, and cohort-specific explanations remain plausible.

However, these findings should be interpreted in the context of several limitations, including the cross-sectional design, incomplete metadata on diet and medication use, and the absence of external validation. Future longitudinal, multi-center, and multi-omics studies will be required to confirm the robustness and generalizability of these ecological patterns and to further elucidate their biological significance.

## Data Availability

The sequencing data are now publicly available and searchable under NCBI BioProject PRJNA1475610. https://www.ncbi.nlm.nih.gov/bioproject/PRJNA1475610.

## Supplementary material

Supplementary Figure 1 Sequencing quality assessment and preprocessing of metagenomic data. **(A)** Comparison of raw reads and high-quality clean reads across the two cohorts, demonstrating comparable sequencing depth among samples. **(B)** Sequencing quality metrics, including Q30 scores and host-derived read proportions. All samples exhibited high sequencing quality (Q30 > 97%) and low host DNA contamination (< 1%), supporting the reliability of downstream microbiome analyses. All samples passed quality-control filtering and were retained for subsequent analyses.

Supplementary Figure 2 Hierarchical clustering of samples based on genus-level microbial abundance profiles.

Supplementary Figure 3 Differentially abundant microbial genera between T2D and control groups Volcano plot showing differential genera between T2D and control groups. Red points indicate genera meeting significance criteria (FDR < 0.05 and |log_2_FC| ≥ 1). All differential abundance analyses were performed using two-sided Wilcoxon rank-sum tests with Benjamini–Hochberg FDR correction unless otherwise specified.

Supplementary Figure 4 Distribution of effect sizes among differentially abundant microbial species. **(A)** Histogram of species-level log_2_ fold changes. **(B)** Density distribution of effect sizes.

Supplementary Figure 5 Volcano plot of pathway-level differential abundance analysis. Gray points indicate pathways not meeting significance criteria (FDR < 0.05 and |log_2_FC| ≥ 1). All differential abundance analyses were performed using two-sided Wilcoxon rank-sum tests with Benjamini–Hochberg FDR correction unless otherwise specified.

Supplementary Figure 6 Differential abundance of branched-chain amino acid biosynthesis pathways.

Supplementary Figure 7 Bile acid metabolism–related pathways exhibit subtle directional shifts in T2D. **(A)** Heatmap of bile acid metabolism–related pathways. **(B)** Comparison of pathway effect sizes.

Supplementary Figure 8 Exploratory partial-effect associations of fasting blood glucose and T2D status with microbial features. Partial-effect analyses showing a negative statistical association between fasting blood glucose and *Bradyrhizobium* assignments and a positive association between T2D status and *Escherichia coli* abundance. The *Bradyrhizobium* signal is technically uncertain, and the *E. coli* association may be confounded by antidiabetic medication exposure.

Supplementary Figure 9 Associations between microbial functional pathways and host clinical variables: Spearman correlation analysis.

Supplementary Figure 10 Associations between microbial functional pathways and host clinical variables: multivariable regression analysis.

Supplementary Figure 11 Performance of pathway-based machine-learning classifiers. **(A)** Feature importance ranking for Random Forest. **(B)** ROC curve for Random Forest. **(C)** Feature importance ranking for XGBoost. **(D)** ROC curve for XGBoost.

Supplementary Figure 12 Consistent contributions of microbial taxa and pathways across machine-learning algorithms. **(A)** Venn diagram showing overlap between the top 10 species ranked by feature importance in the Random Forest and XGBoost models. Nine species were shared between the two models, with one species unique to each model. **(B)** Venn diagram showing overlap between the top 10 microbial pathways ranked by feature importance in the Random Forest and XGBoost models. Nine pathways were shared between the two models, with one pathway unique to each model.

Supplementary Figure 13 SHAP summary plot for the Random Forest model.

Supplementary Figure 14 SHAP summary plot for the XGBoost model.

Supplementary Table 1 Clinical and demographic characteristics of study participants.

Supplementary Table 2 Differential abundance analysis of microbial genera between T2D and healthy controls.

Supplementary Table 3 Differential abundance analysis of microbial species between T2D and healthy controls.

Supplementary Table 4 Differential abundance analysis of microbial metabolic pathways.

Supplementary Table 5 Comparative analysis of bile acid metabolism–related pathways.

Supplementary Table 6 Significant Spearman correlations between microbial species and host clinical variables.

Supplementary Table 7 Multivariable microbial association analysis adjusted for age, BMI, fasting blood glucose, and T2D status.

Supplementary Table 8 Spearman correlations between microbial pathways and host clinical variables.

Supplementary Table 9 Multivariable association analysis of microbial pathways and host phenotypes.

Supplementary Table 10 Enterotype assignment and association with T2D status.

Supplementary Table 11 Feature importance scores and SHAP values from taxonomic and functional machine-learning models.
