# Supplementary material for: Multi-layer gut microbiome variation in type 2 diabetes despite preserved higher-order community structure
Source: Front Microbiol. 2026 Aug 14;17:1902019. doi: 10.3389/fmicb.2026.1902019 (PMC13522148; doi:10.3389/fmicb.2026.1902019)

Supplementary Figure 1

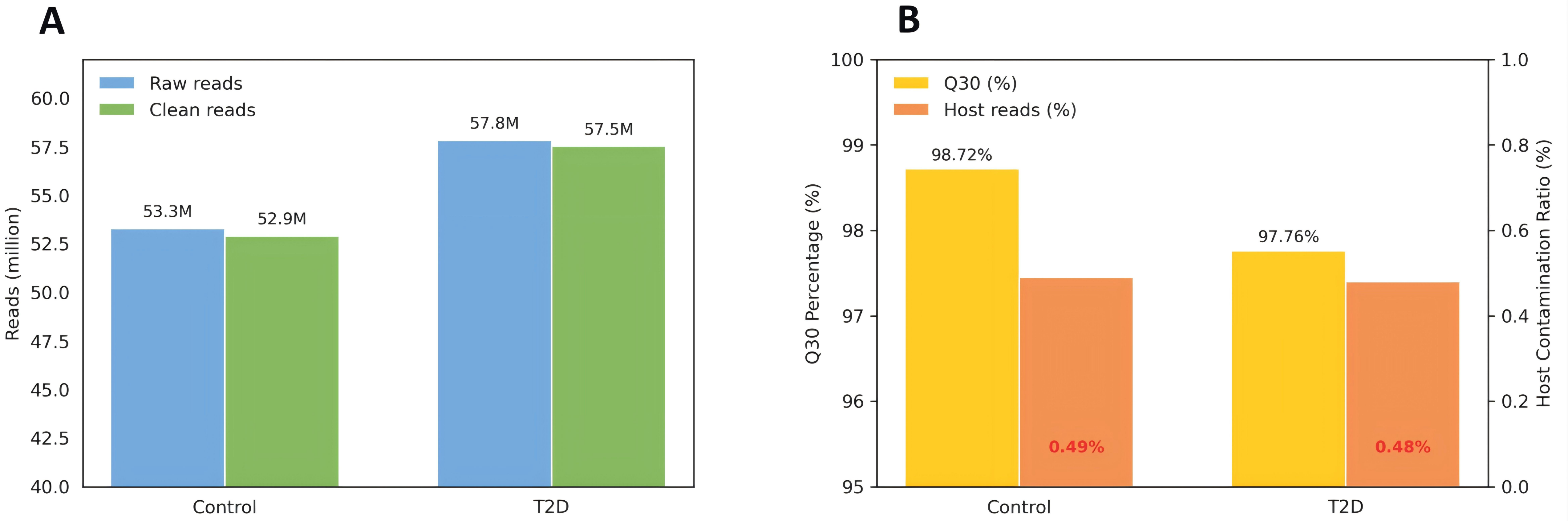

Supplementary Figure 2

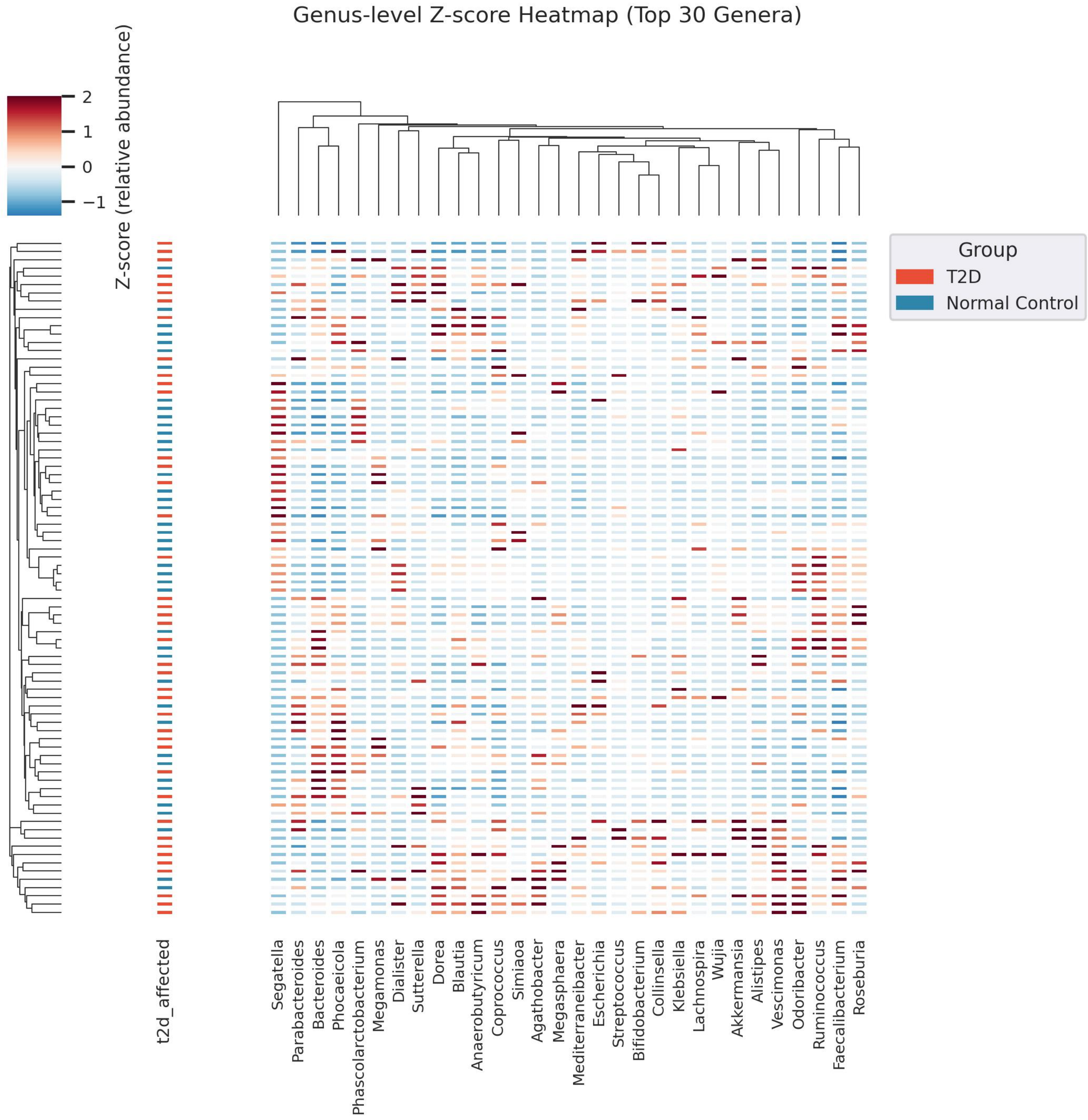

Supplementary Figure 3

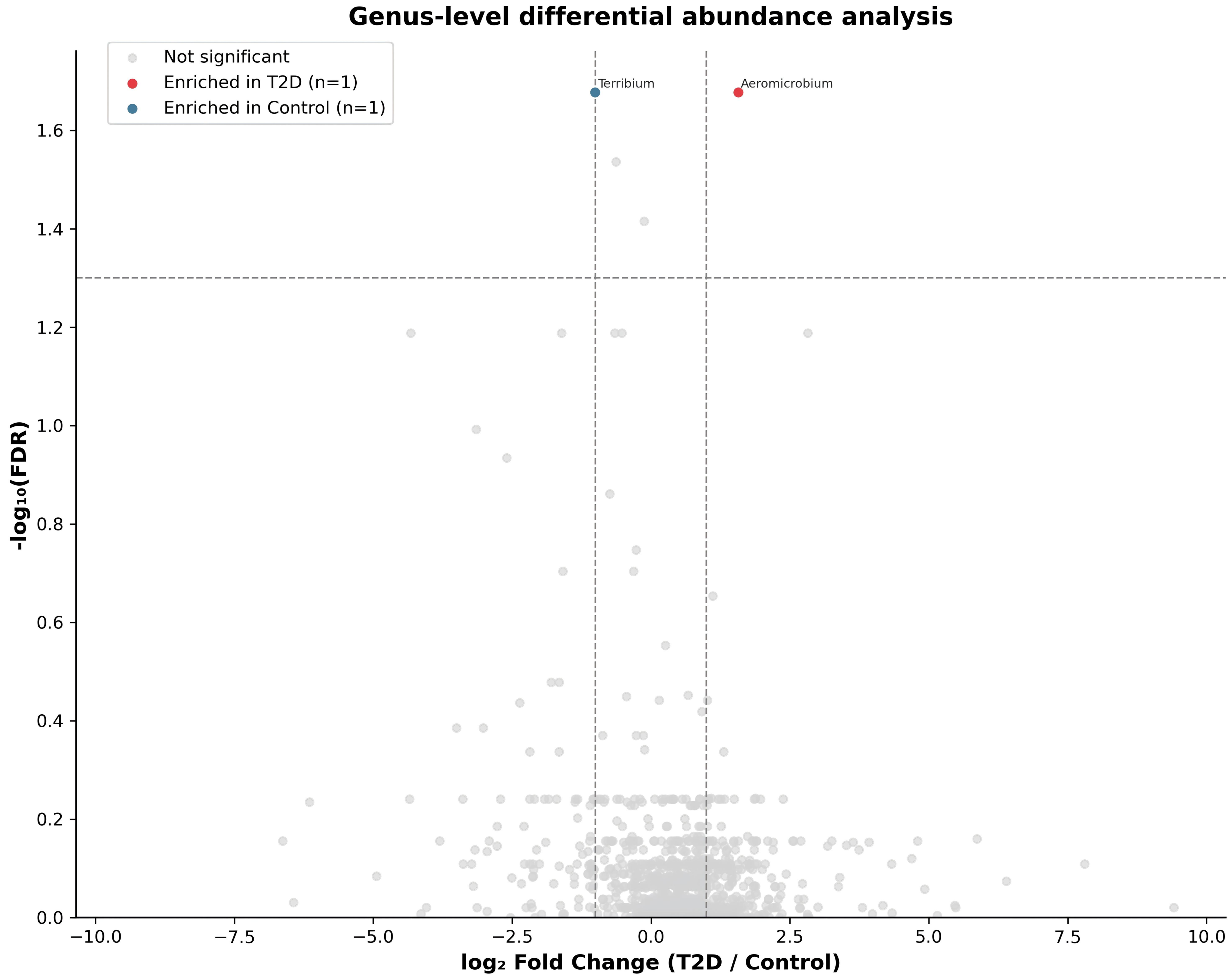

Supplementary Figure 4

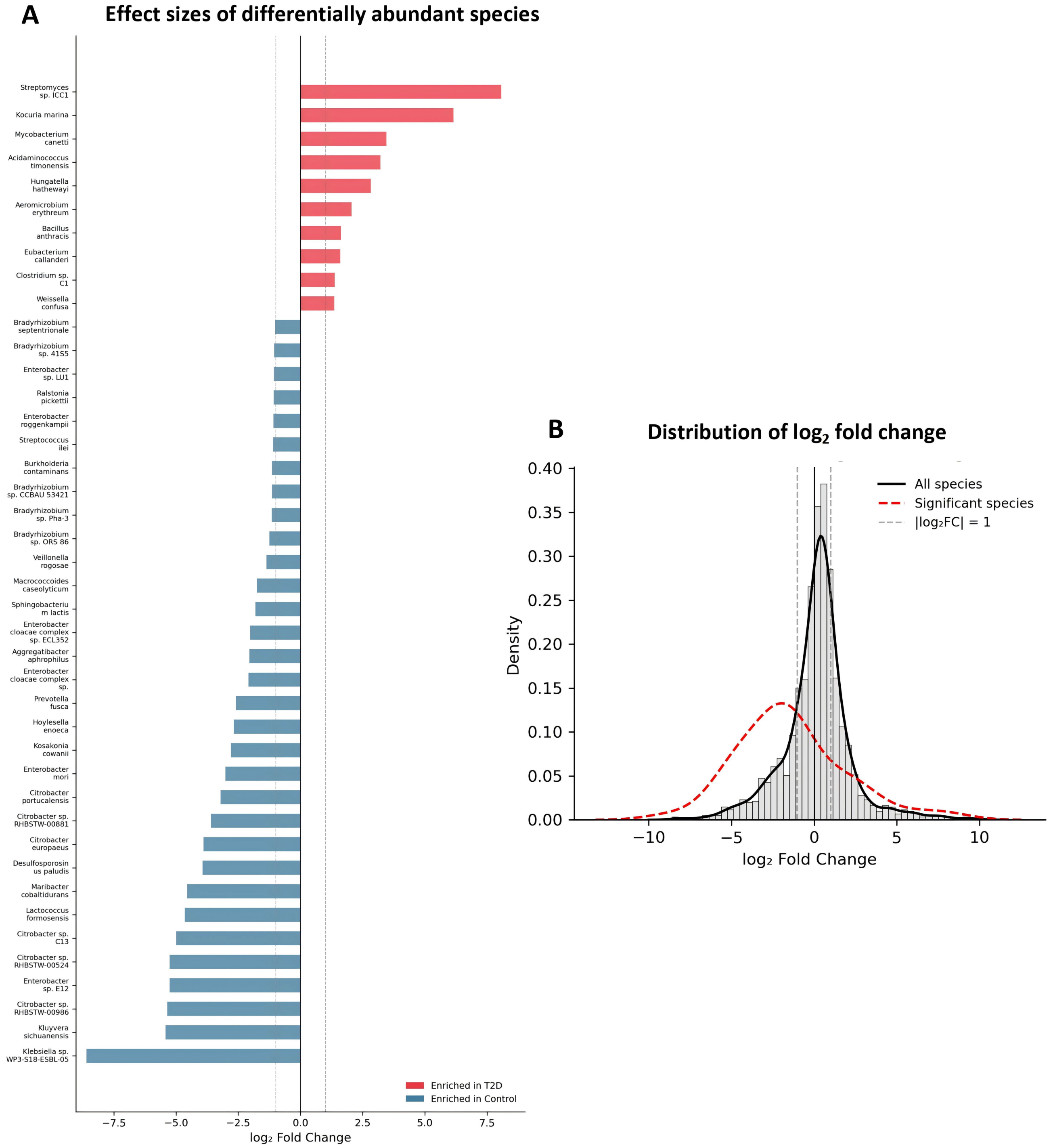

Supplementary Figure 5

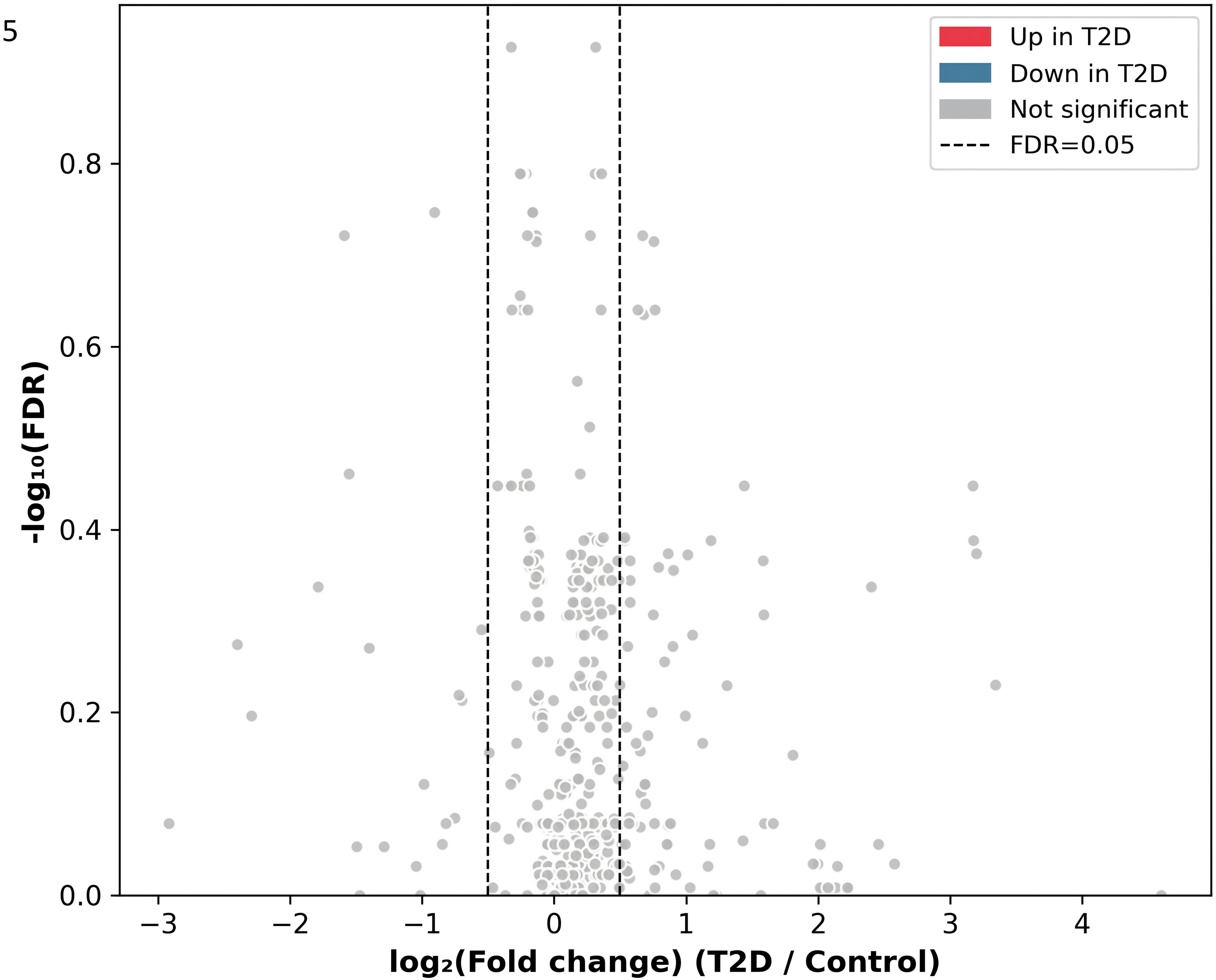

Supplementary Figure 6

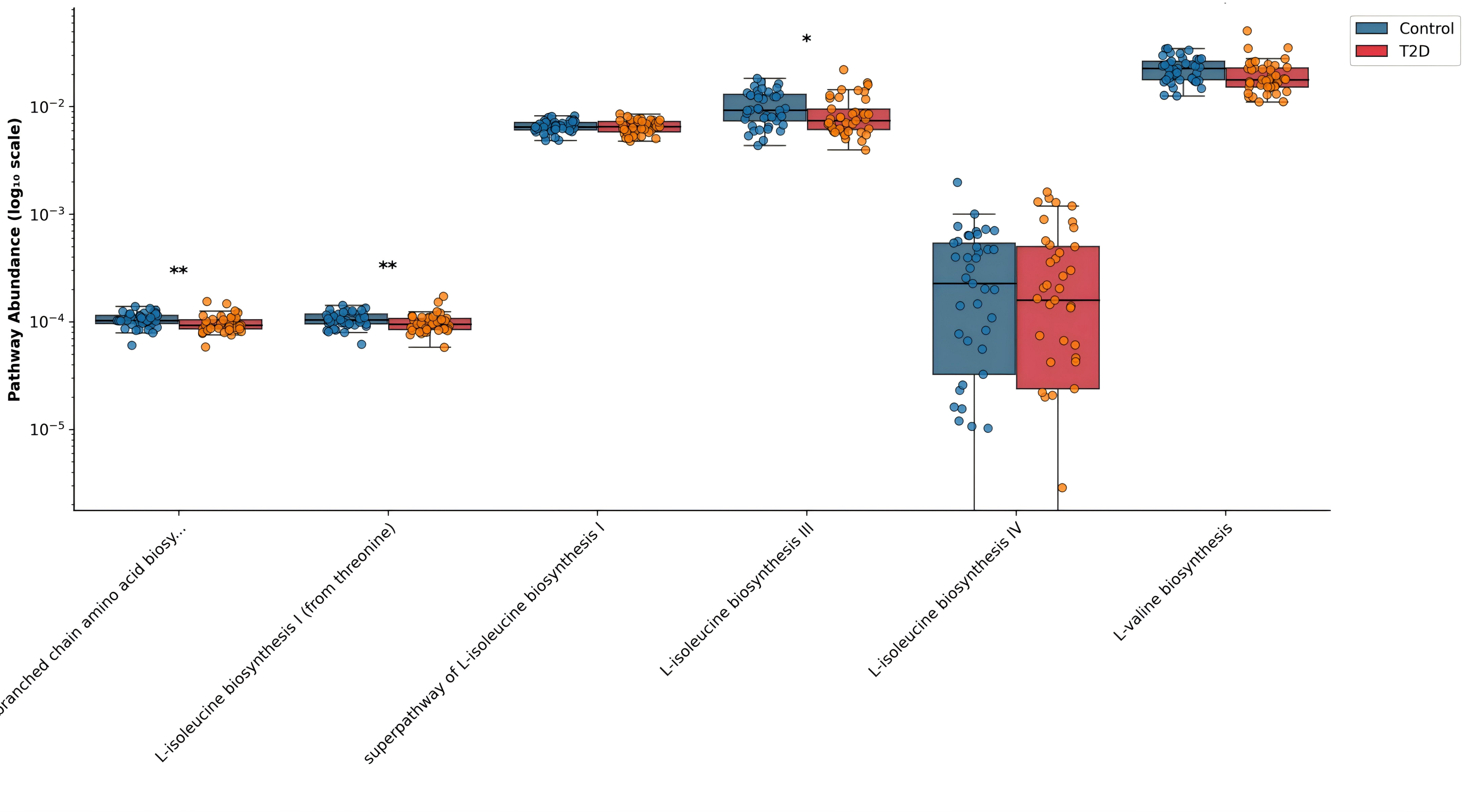

Supplementary Figure 7

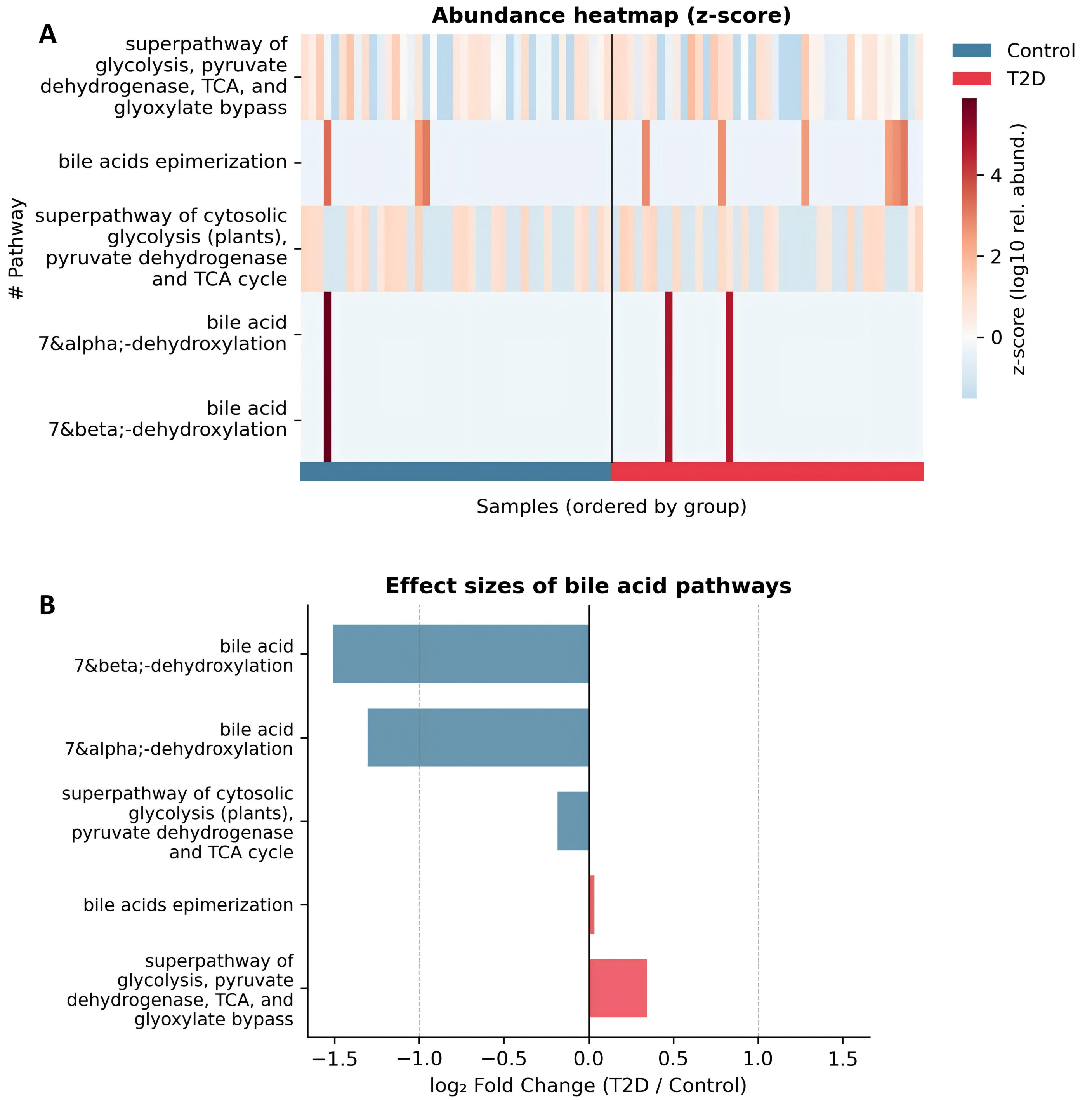

Supplementary Figure 8

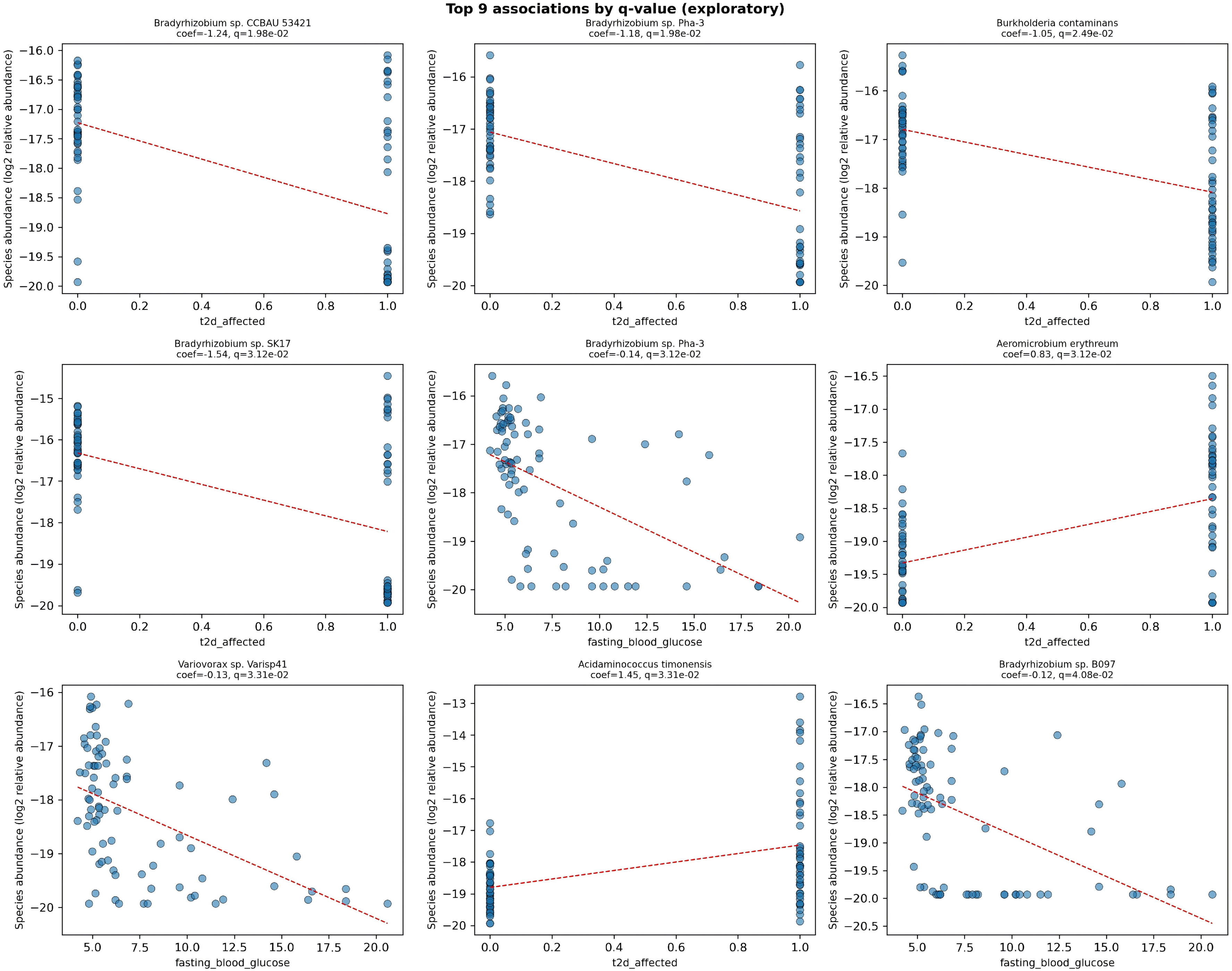

Supplementary Figure 9

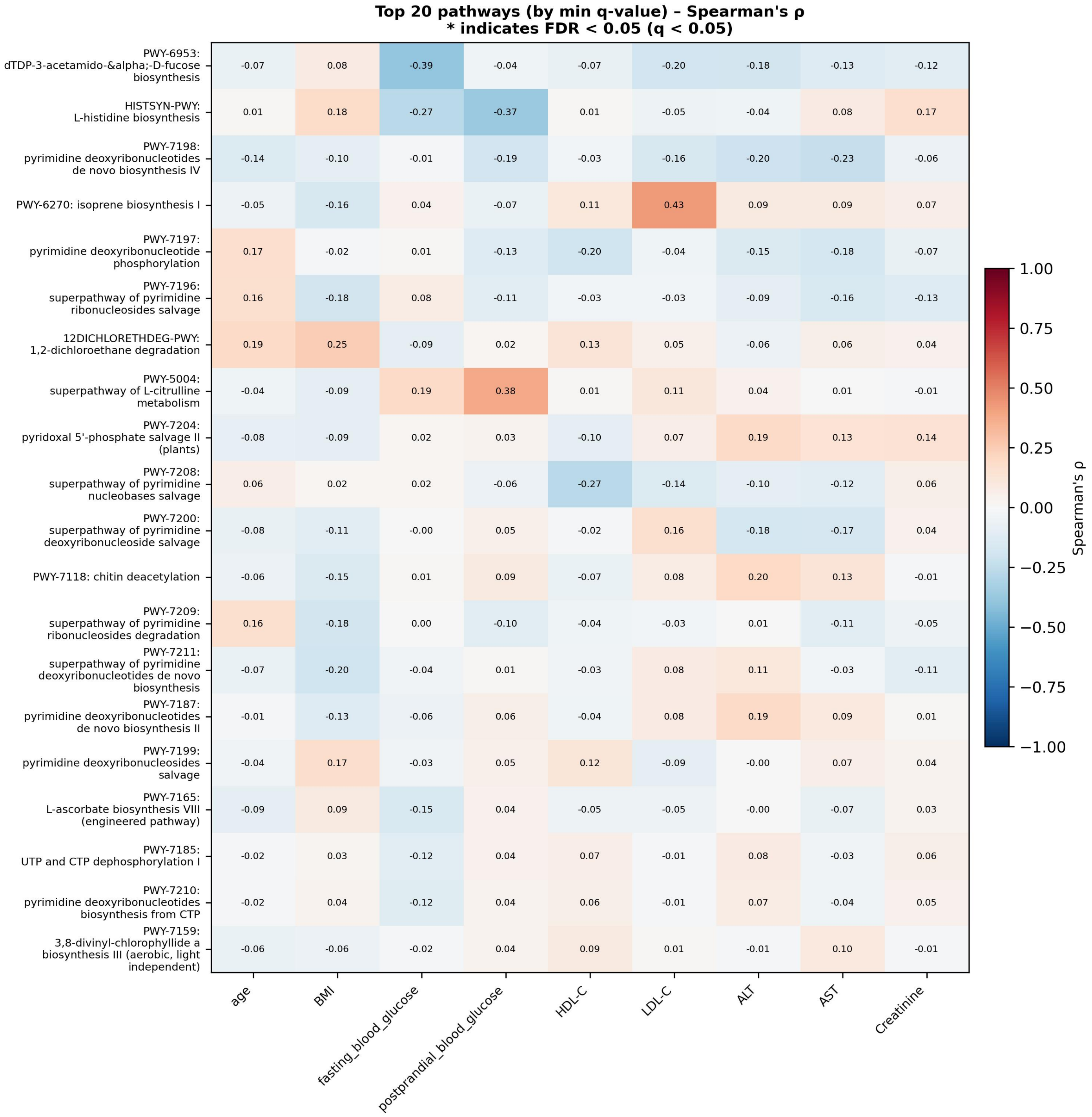

Supplementary Figure 10

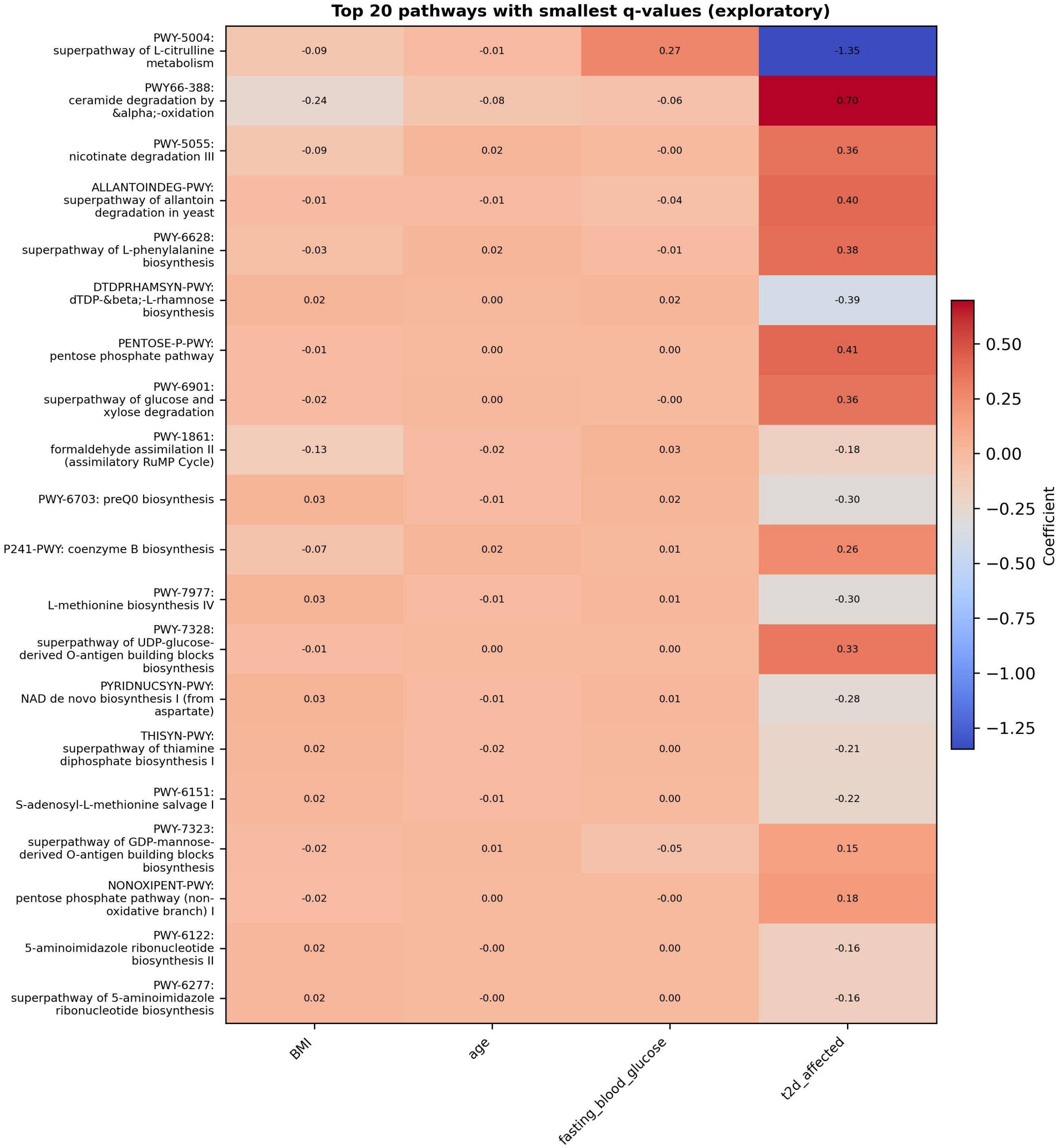

Supplementary Figure 11

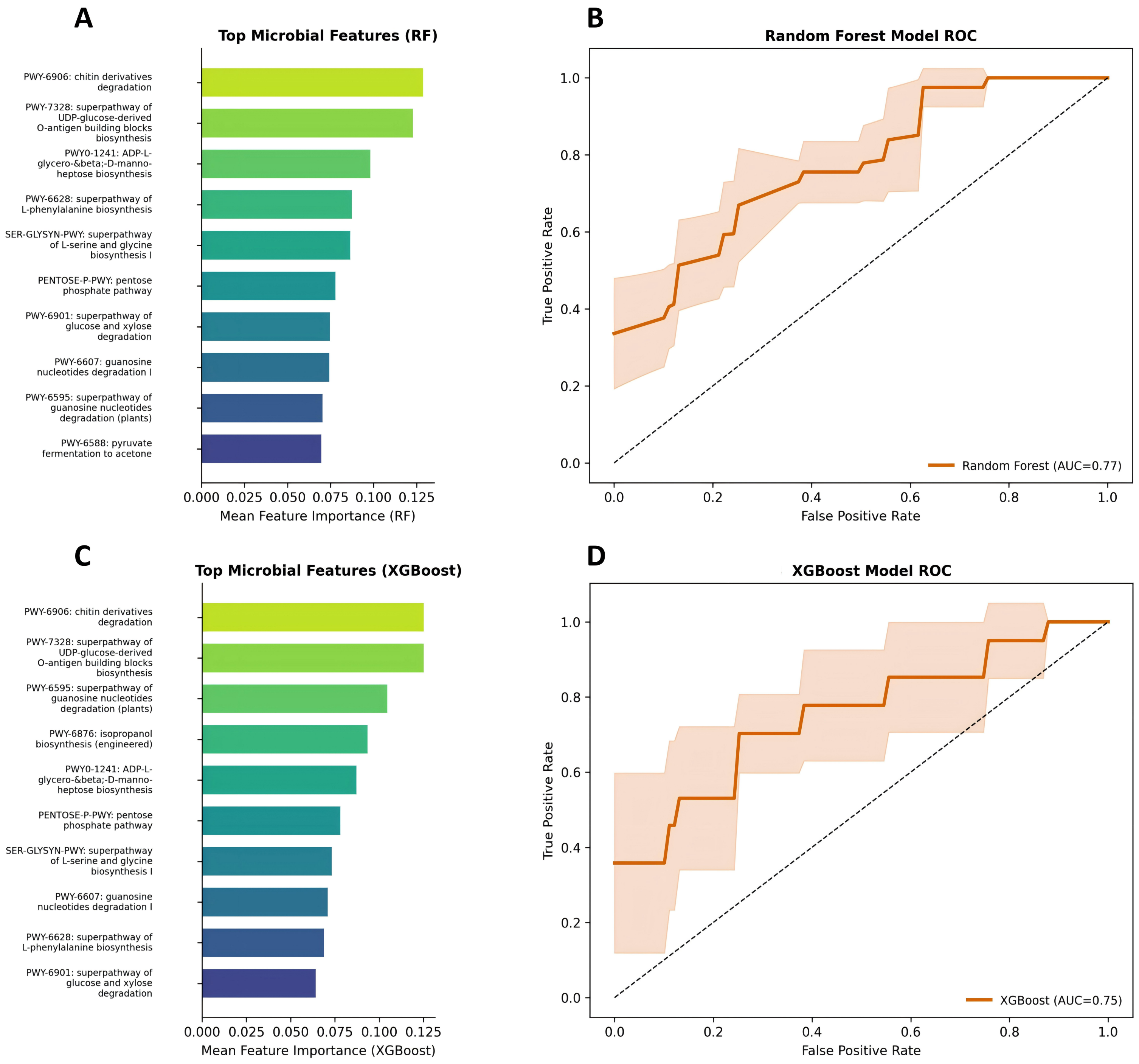

Supplementary Figure 12

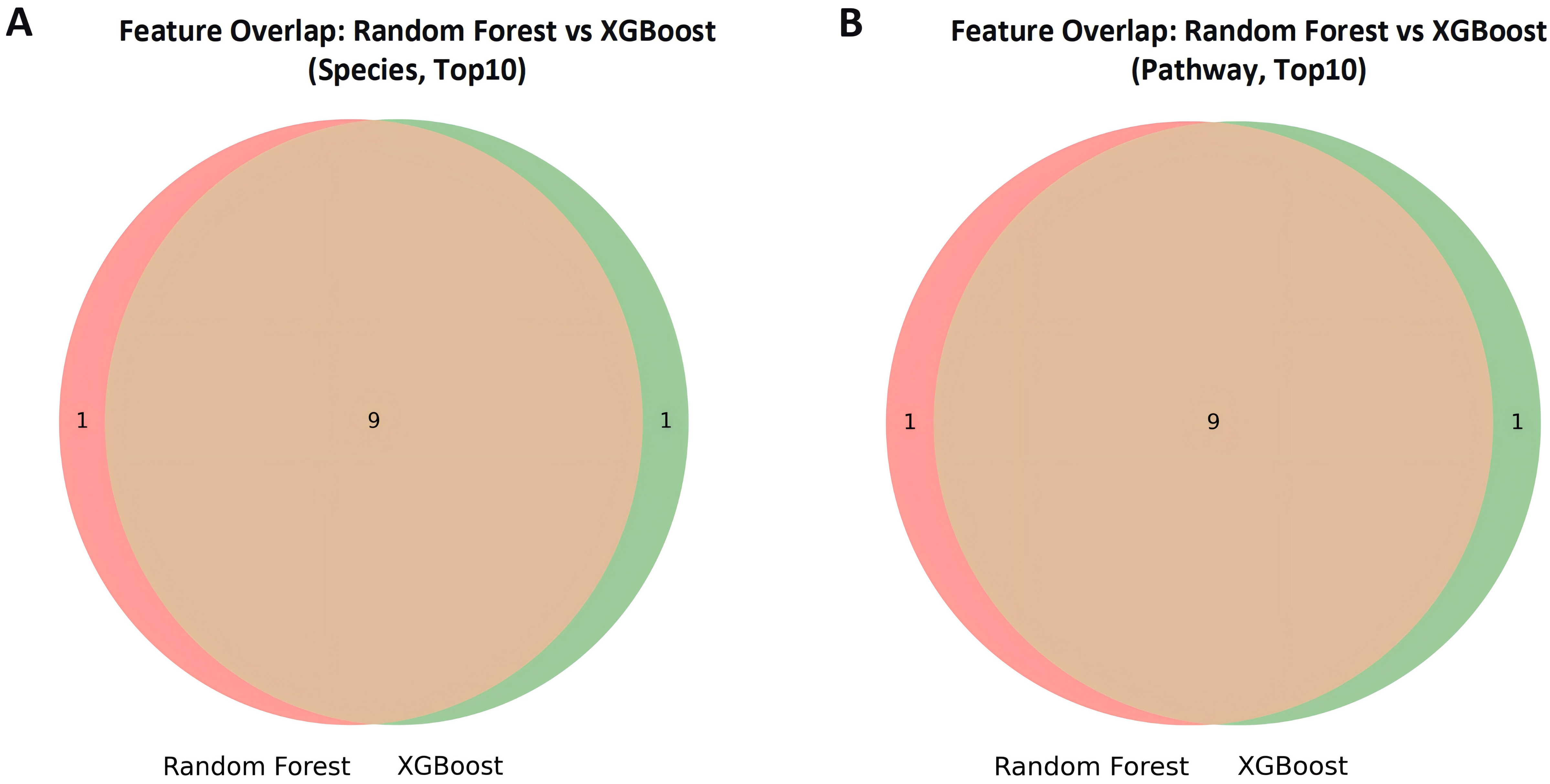

Supplementary Figure 13

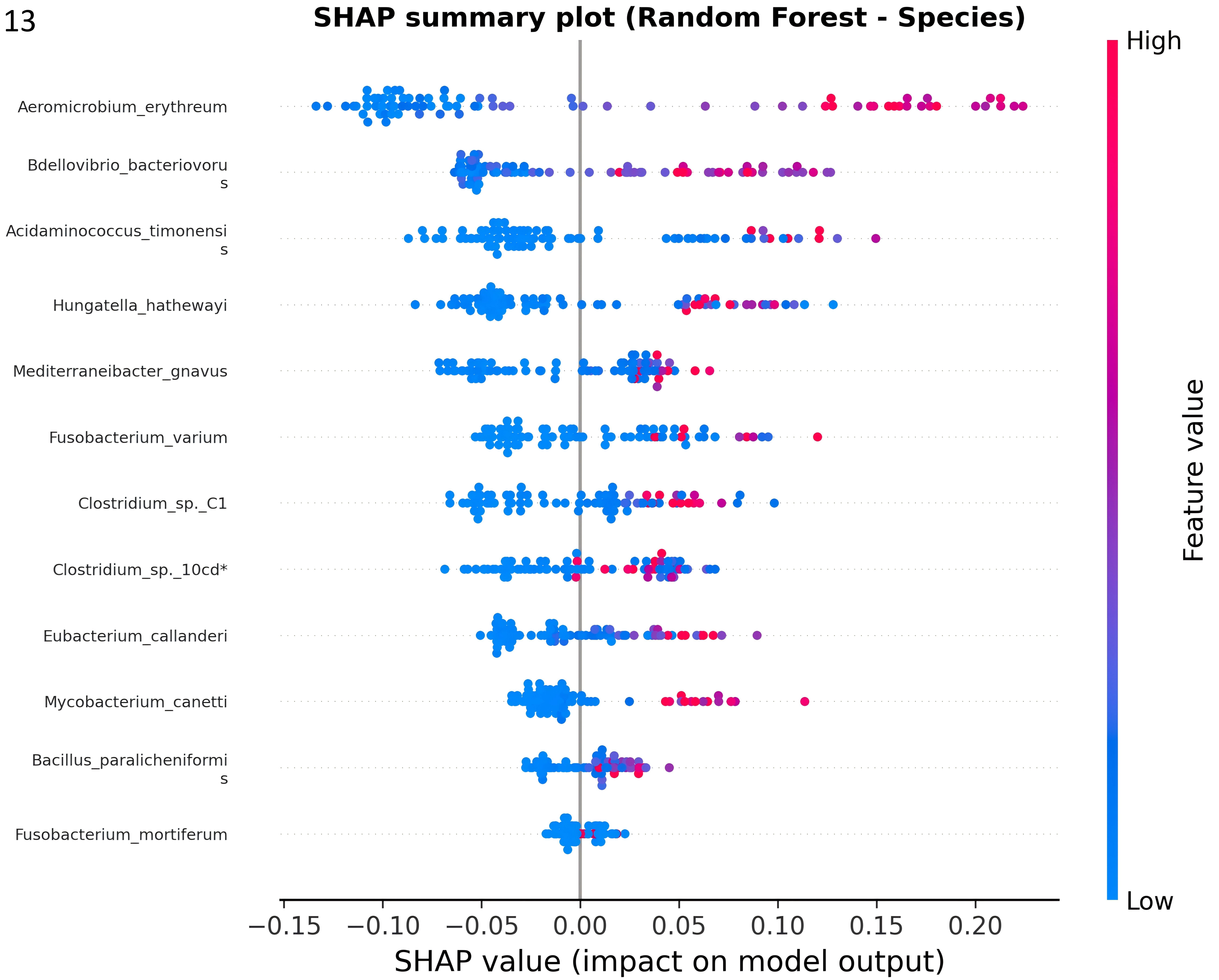

Supplementary Figure 14

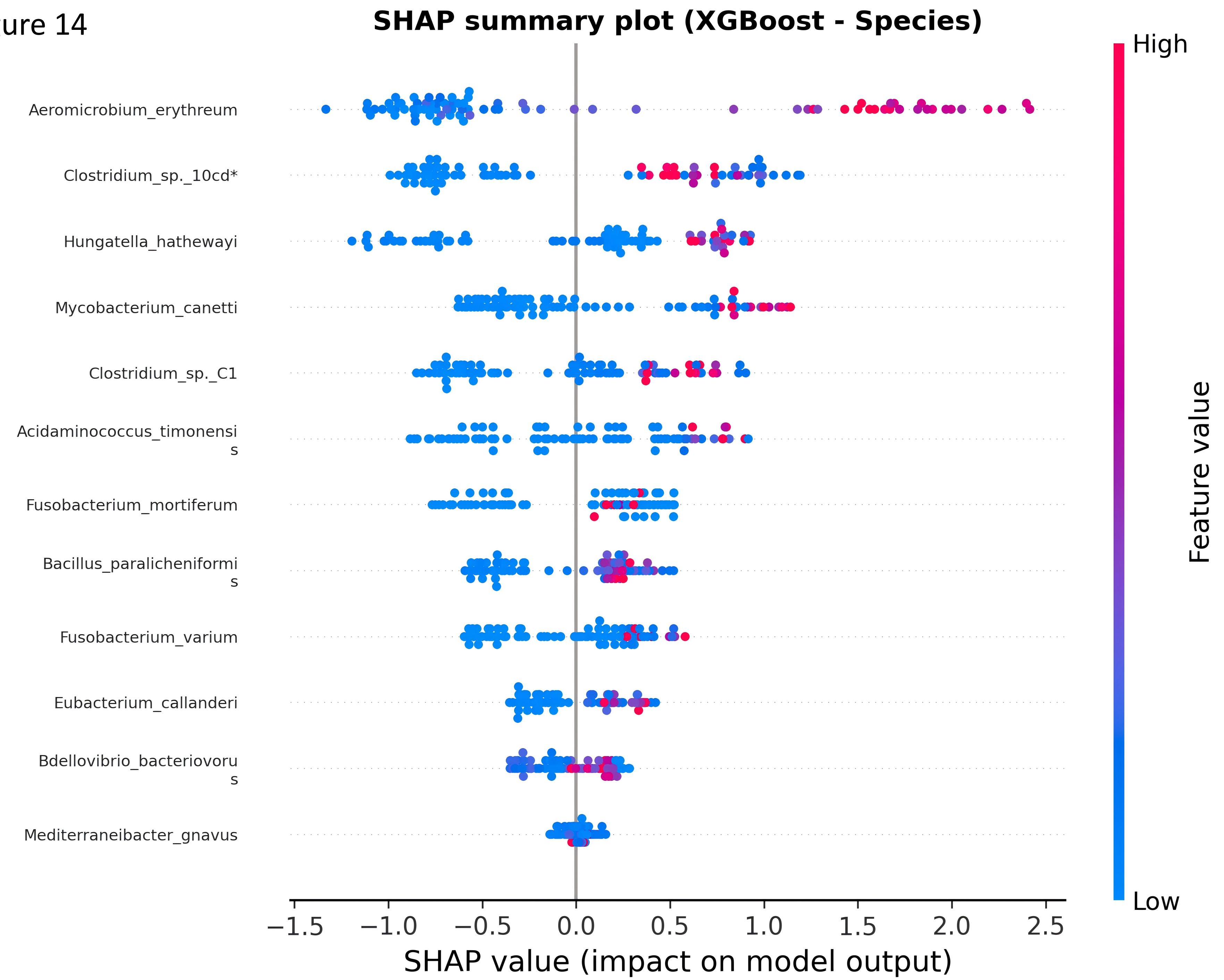

Supplement: Supplementary file 1 [file Data_Sheet_1.pdf]
